# Postoperative absence of residual intracranial tumor volume is associated with improved survival and intracranial disease control in non-small cell lung cancer brain metastases

**DOI:** 10.1007/s11060-025-05387-1

**Published:** 2025-12-22

**Authors:** Jacopo Bellomo, Meltem Gönel, Jonathan Sutter, Anna Maria Zeitlberger, Maria Nikolaeva, Tristan Schmidlechner, Vittorio Stumpo, Luis Padevit, Victor Egon Staartjes, Flavio Vasella, Jorn Fierstra, Nicolin Hainc, Isabelle Opitz, Michael Weller, Emilie Le Rhun, Oliver Bozinov, Luca Regli, Carlo Serra, Marian Christoph Neidert, Stefanos Voglis

**Affiliations:** 1 Department of Neurosurgery, Clinical Neuroscience Center, University Hospital and University of Zurich, Frauenklinikstrasse 10, Zurich, 8091 Switzerland; 2 Department of Neurosurgery, HOCH Health Ostschweiz, St. Gallen, Switzerland; 3 Department of Neuroradiology, Clinical Neuroscience Center, University Hospital and University of Zurich, Zurich, Switzerland; 4 Department of Thoracic Surgery, University Hospital and University of Zurich, Zurich, Switzerland; 5 Department of Neurology, Clinical Neuroscience Center, University Hospital and University of Zurich, Zurich, Switzerland

**Keywords:** Lung cancer, Brain metastasis, Residual tumor, Extent of resection, Immunotherapy, Targeted therapy, Overall survival, Progression-free survival

## Abstract

**Background:**

Brain metastases (BM) from non-small cell lung cancer (NSCLC) are associated with limited prognosis. Although surgical resection is part of multimodal management, the prognostic relevance of surgical intracranial tumor load reduction in the era of stereotactic radiotherapy and modern systemic therapies remains unclear.

**Methods:**

This retrospective bicentric cohort study included 285 adults with histologically confirmed NSCLC who underwent BM resection. Pre- and postoperative MRI were used for volumetric assessment. Gross-total resection (GTR) was defined as the absence of measurable postoperative residual volume (RV). Outcomes were overall survival (OS) and intracranial progression-free survival (iPFS). Multivariable Cox regression models adjusted for age, preoperative performance status, extracranial metastases, number of BM, and postoperative radiotherapy and systemic therapies.

**Results:**

Median OS was 14.3 months and median iPFS was 8.0 months. GTR was achieved in 96 patients (34% overall; 38% of patients with evaluable volumetric imaging) and was independently associated with longer OS (adjusted HR, 0.51; 95% CI, 0.30–0.86) and prolonged iPFS (adjusted HR, 0.55; 95% CI, 0.31–0.99). Postoperative RV, analyzed continuously or categorically, showed no consistent association with OS or iPFS. The benefit of GTR was most pronounced in patients with single BM or without extracranial metastases.

**Conclusion:**

Achieving complete intracranial tumor resection, rather than the amount of postoperative residual volume, was consistently associated with improved survival and intracranial disease control. These findings support the clinical relevance of GTR in selected NSCLC-BM patients and may inform patient counselling and surgical decision-making, while warranting further investigation in patients with multifocal but safely resectable intracranial disease.

**Supplementary Information:**

The online version contains supplementary material available at 10.1007/s11060-025-05387-1.

## Introduction

Lung cancer is the most frequent primary malignancy leading to brain metastases (BM), with up to 40% of patients developing intracranial tumor manifestations during the disease course [1–3]. Despite recent advances in systemic therapies – particularly immune checkpoint inhibitors (ICI) and targeted molecular therapies – BM remain associated with substantial morbidity and a dismal prognosis [4–6].

Surgical resection of BM remains an essential treatment pillar in selected patients, particularly those with a limited number or symptomatic lesions, accessible tumor location, and preserved functional status [7, 8]. Beyond symptom relief, surgey provides tissue for histological and molecular characterization, which may guide subsequent systemic treatment decisions [8]. However, the prognostic relevance of intracranial tumor burden reduction – quantified by gross total resection (GTR), extent of resection (EOR), or postoperative residual tumor volume (RV) – remains controversial.

Prior studies have reported inconsistent associations between GTR or residual volume and survival in patients with BM [9], often limited by heterogeneous cohorts including different primary tumor entities, incomplete imaging-based assessment of EOR, or inadequate adjustment for various confounding factors including systemic therapies. A recent systematic review and meta-analysis across BM subtypes, confirmed an overall association between GTR and improved OS, while emphasizing the need for tumor entity-specific evidence and adjustment for other treatment regiments to guide neurosurgical resection strategies in BM patients [9].

Given the high incidence of BM in lung cancer patients and the increasing efficacy of systemic therapies, a better understanding of the prognostic relevance of surgical tumor burden reduction in this population is warranted. In this bicentric study, we retrospectively analyzed patients with non-small cell lung cancer BM (NSCLC-BM) who underwent neurosurgical resection. We aimed to investigate whether intracranial tumor burden reduction – measured by GTR and postoperative RV – is associated with OS and iPFS, both in the overall cohort and within predefined subgroups.

## Methods

### Study cohort, data acquisition and ethical considerations

This retrospective observational study was conducted at two tertiary brain tumor centers in Switzerland: the Department of Neurosurgery at the University Hospital Zurich (USZ) and the Department of Neurosurgery at the Cantonal Hospital St. Gallen (KSSG). All consecutive adult patients who underwent a first neurosurgical resection of intraparenchymal BM from histologically confirmed primary non-small-cell lung cancer (NSCLC) between December 2011 and January 2022 at USZ, and between April 2013 and March 2022 at KSSG, were included. Patients with prior surgical treatment of brain metastases were excluded from the entire cohort. Patients with insufficient pre- or postoperative MRI were excluded only from volumetric analyses (see section “Volumetric analysis”) but were retained in descriptive analyses of the overall surgically treated cohort. Indications for neurosurgical resection were determined by multidisciplinary tumor boards according to contemporary international guidelines and included symptomatic lesions with mass effect, large metastases not amenable to stereotactic radiotherapy, and the need for histopathological diagnosis [8]. Surgical resection was generally limited to the dominant or symptomatic metastasis, with multiple-lesion resections performed only in selected cases. Patients were managed within a multidisciplinary framework according to contemporaneous standards of care, including postoperative radiotherapy (stereotactic radiotherapy, SRT, or, in selected cases, whole-brain radiotherapy, WBRT) and evaluation for systemic therapies such as chemotherapy, immunotherapy, or targeted therapy (TT). Postoperative stereotactic radiotherapy followed evidence-based institutional standards. Radiotherapy and systemic therapy classification reflected all postoperative treatments delivered during the disease course, rather than only the initial adjuvant modality.

Clinical outcomes included OS, defined as the time from BM surgery to death or last follow-up, and iPFS, defined as the time from surgery to radiologically confirmed local or distant intracranial disease progression, including leptomeningeal dissemination, as confirmed by a board-certified neuroradiologist and documented in tumor board reports.

This study was approved by the local ethics committees of both institutions (KEK-ZH-Nr. 2017–00093; EKOS-Nr. 2023–01343) and conducted in accordance with the Declaration of Helsinki. Written informed consent was obtained from all participants.

### Volumetric analysis

Pre- and postoperative (within 72 h) native and contrast-enhanced T1-weighted magnetic resonance imaging (MRI) sequences were reviewed by two experienced neurosurgeons (S.V. and M.N.) with the help of neuroradiological reports to determine total number of BM and the total intracranial tumor volume. Tumor volumes (in cubic centimeters, cm^3^) were semi-automatically segmented on T1-weighted gadolinium-enhanced images, using iPlan Net (Brainlab AG, Munich, Germany). The EOR was calculated as: [1 - (postoperative total intracranial tumor volume of all residual lesions/preoperative total intracranial tumor volume) x 100]. GTR was defined as the 100% removal of the total intracranial tumor volume. The postoperative RV was defined as the total intracranial remaining contrast-enhancing tumor burden following resection.

### Statistical analysis

All statistical analyses were performed using R (version 4.3.1) and RStudio (version 2023.09.1) with open-source libraries. Descriptive statistics were computed, including frequencies, medians with interquartile ranges (IQR), and means with standard deviations (SD) where appropriate. Survival functions were estimated using Kaplan-Meier analysis, with differences between groups compared using the log-rank test. Univariable and multivariable Cox proportional hazards regression models were applied to evaluate associations between GTR, postoperative RV, and survival outcomes. Because postoperative RV exhibited a markedly right-skewed distribution, values were log-transformed prior to modeling. Multivariable models adjusted for age at BM surgery, preoperative ECOG status, the presence of visible extracranial metastases at time of surgery, number of BM, presence of neurological symptoms or signs related to the BM, postoperative radiotherapy including SRT and WBRT, as well as postoperative systemic therapy including ICI and TT. Predefined subgroup analyses were conducted. Specifically, we stratified patients by (1) number of BM at time of surgery, (2) presence of extracranial metastases at time of surgery, (3) postoperative ICI therapy, and (4) postoperative TT. Within each subgroup, the associations of GTR and RV with OS and iPFS were reassessed using stratified Cox regression models.

Missing values were handled by case-wise deletion, assuming data were missing at random. Two-sided p-values < 0.05 were considered statistically significant.

## Results

### Study cohort and clinical characteristics

A total of 285 patients with NSCLC-BM who underwent neurosurgical resection were included in the overall cohort. Postoperative volumetric analyses were feasible in 232 patients due to pre- and postoperative MRI availability. Baseline demographic, clinical, and treatment characteristics are summarized in Table 1 and illustrated in Supplemental Fig. 1. The median age at the time of surgery was 64.0 years [IQR, 56.0-68.9], and 146 patients (51.2%) were male. A total of 145 patients (50.8%) presented with synchronous BM (diagnosis within 2 months from lung cancer diagnosis), while 126 patients (44.2%) had metachronous BM (diagnosis more than 2 months after lung cancer diagnosis). In 14 patients (4.9%), this information was unavailable due to missing data. At presentation, 250 patients (87.7%) had neurological symptoms or signs attributable to BM. Preoperative ECOG performance status was ≤ 1 in 60.3% of cases (*n* = 172). Extracranial metastases were present at the time of BM resection in 137 patients (48.1%). The distribution of the number of BM was as follows: 1 lesion in 155 patients (54.4%), 2–5 lesions in 98 patients (34.4%), and more than 5 lesions in 32 patients (11.2%). Postoperatively, 232 patients (81.4%) received radiotherapy (152 patients received only SRT, 46 patients only WBRT, and 34 patients both), 46 patients (16.1%) received TT, and 88 (30.9%) received immunotherapy.

**Table 1:** Study cohort characteristics

	*N* = 285
Age at BM surgery, median [IQR]	64 [56-69.8]
Sex (%)	Males: 146 (51.2) Females: 139 (48.8)
Preoperative ECOG status (%)	0: 48 (16.8) 1: 124 (43.5) 2: 31 (10.9) 3: 13 (4.6) 4: 1 (0.4) NA: 68 (23.9)
BM symptoms and signs (%)	Asymptomatic: 35 (12.3) Symptomatic: 250 (87.7)
EGFR mutation status of primary tumor (%)	Negative: 79 (27.7) Positive: 12 (4.2) NA: 194 (68.1)
ALK mutation status of primary tumor (%)	Negative: 82 (28.8) Positive: 4 (1.4) NA: 199 (69.8)
PD-L1 expression status of primary tumor (%)	TC0: 24 (8.4) TC1: 8 (2.8) TC2: 5 (1.8) TC3: 4 (1.4) NA: 244 (85.6)
Time (in months) between LC and BM diagnosis, median [IQR]	0.8 [0-13.9]
Time (in days) between BM diagnosis and DOS, median [IQR]	7.5 [4–22]
Extracranial metastatic lesion(s) at BM diagnosis (%)	No: 140 (49.1) Yes: 137 (48.1) NA: 8 (2.8)
Number of BM (%)	1: 155 (54.4) 2–5: 98 (34.4) More than 5: 32 (11.2)
Preoperative intracranial tumor volume [cm^3^], mean (SD)	17.34 (19.31)
Preoperative radiotherapy (%)	STR: 22 (7.7) WBRT: 3 (1.1)
Preoperative immunotherapy (%)	No: 193 (67.7) Yes: 33 (11.6) NA: 59 (20.7)
Preoperative TT (%)	No: 216 (75.8%) Yes: 10 (3.5%) NA: 59 (20.7%)
EOR, mean (SD)	88.65 (21.19)
GTR (%)	Achieved: 96 (33.7) Not achieved: 189 (66.3)
Postoperative intracranial tumor volume [cm^3^], mean (SD)	1.93 (5.31)
Postoperative radiotherapy (%)	SRT: 152 (53.3) WBRT: 46 (16.1) Both: 34 (11.9)
Postoperative immunotherapy (%)	No: 136 (47.7%) Yes: 88 (30.9%) NA: 61 (21.4%)
Postoperative TT (%)	No: 177 (62.1%) Yes: 46 (16.1%) NA: 62 (21.8%)
Follow-up time (in months), median [IQR]	13.1 [5.1–29.6]

ALK, anaplastic lymphoma Kinase; BM, brain metastasis; DOS, day of surgery; ECOG, Eastern cooperative oncology Group; EGFR, epidermal growth factor Receptor; EOR, extent of resection; GTR, gross total resection (defined as EOR = 100%); IQR, interquartile range; LC, lung cancer; NA, not available; SD, standard deviation; SRT, stereotactic radiotherapy; TT targeted therapy; TC, tumor cell score (TC0: <1%; TC1: 1–4%; TC2: 5–49%; TC3: ≥50%) [31]; WBRT, whole-brain radiotherapy

### Pre- and postoperative total intracranial tumor load

The mean preoperative intracranial tumor volume was 17.34 cm³ (SD, 19.31), and the mean postoperative RV was 1.93 cm³ (SD, 5.31). The mean EOR, relative to all intracranial lesions, was 88.65% (SD, 21.19). Among patients with available volumetric data, GTR (i.e., 100% EOR) was achieved in 96 of 253 patients (38%). GTR was achieved in 85 of 126 patients (67%) with a single brain metastasis and in 11 of 106 patients (10%) with multiple brain metastases. Supplemental Fig. 2 illustrates the paired preoperative and postoperative tumor volumes stratified by the number of BM (1 vs. >1) and displaying the individual patient-level EORs. Supplemental Fig. 3 shows the distribution of postoperative intracranial residual tumor volume stratified by number of brain metastases.

### Overall survival analysis

**Fig. 1:**
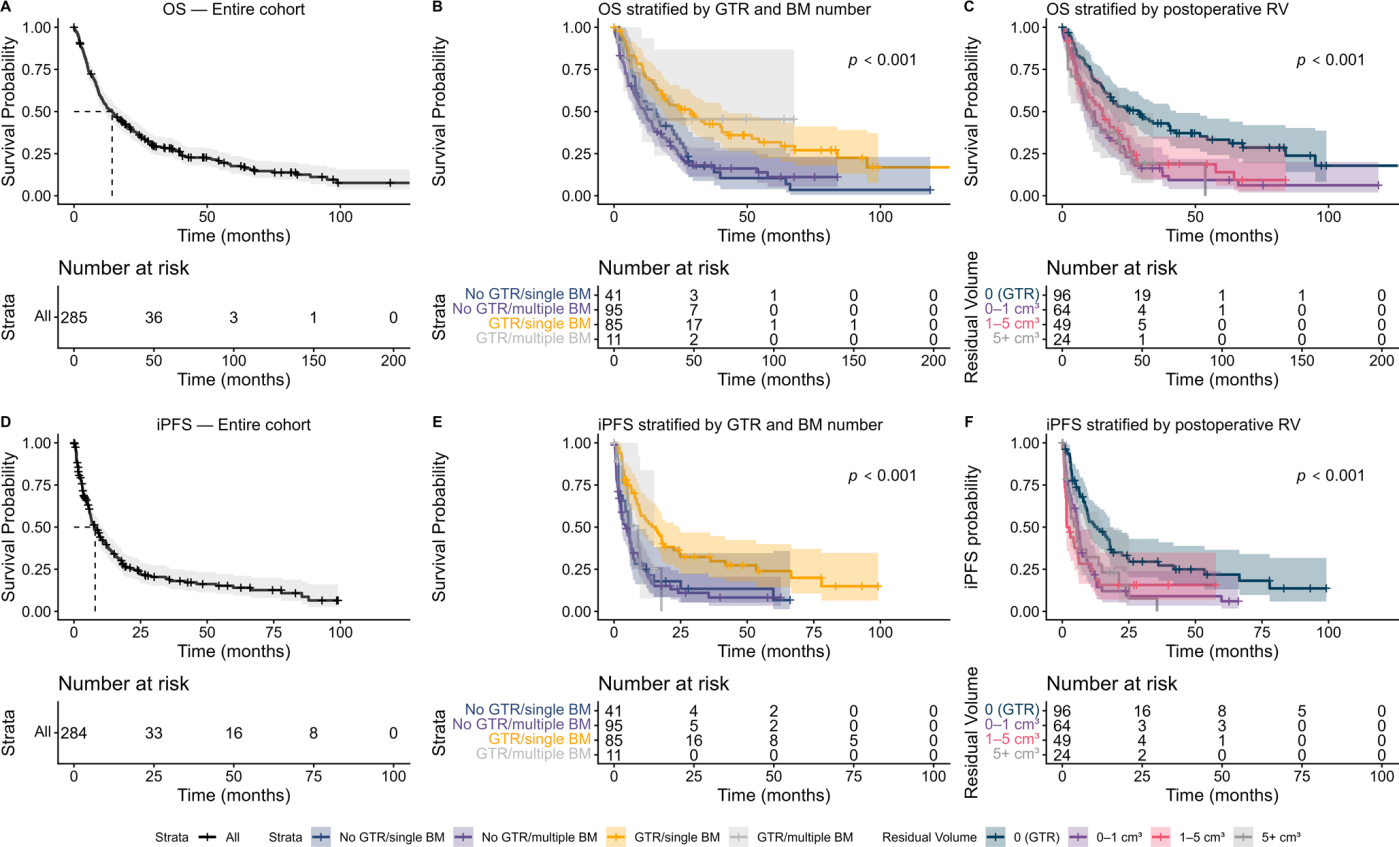
Kaplan–Meier curves of overall and intracranial progression-free survival of the study cohort. (**A**) Overall survival (OS) of the entire cohort (*n* = 285). (**B**) OS stratified by achievement of gross-total resection (GTR) and the number of brain metastases (BM) at time of surgery (single versus multiple; *n* = 253). (**C**) OS stratified by postoperative residual tumor volume (RV) categories (0 cm^3^ [GTR], > 0–1 cm^3^, > 1–5 cm^3^, > 5 cm^3^; *n* = 253). (**D**) Intracranial progression-free survival (iPFS) of the entire cohort (*n* = 284; 1 patient with missing data with respect to local recurrence). (**E**) iPFS stratified by achievement of GTR and the number of BM at time of surgery (single versus multiple; *n* = 253). (**F**) iPFS stratified by postoperative RV categories (0 cm^3^ [GTR], > 0–1 cm^3^, > 1–5 cm^3^, > 5 cm^3^; *n* = 253). Numbers at risk are shown below each survival curve. Shaded areas indicate 95% confidence intervals

The median OS for the entire cohort was 14.3 months (95%CI, 11.1–18.4; Fig. 1A). In univariable analysis (Table 2), both GTR (HR 0.50; 95%CI, 0.36–0.68; *p* < 0.001) and lower postoperative RV (HR 1.18; 95%CI, 1.04–1.34; *p* = 0.010) were associated with longer OS.

**Table 2:** Cox regression analysis of prognostic factors for overall survival

	Univariable		Multivariable			
	HR (95%CI)	*P* value	HR (95%CI)	*P* value	HR (95%CI)	*P* value
**Gross-total resection (GTR)**						
- No	-					
- Yes	0.50 (0.36–0.68)	< 0.001*	0.51 (0.30–0.86)	0.012*		
**Postoperative residual tumor volume [cm** ^**3**^ **]#**	1.18 (1.04–1.34)	0.010*			1.14 (0.88–1.47)	0.31
**Age at BM surgery**	1.03 (1.02–1.05)	< 0.001*	1.03 (1.00-1.05)	0.038*	1.03 (1.00-1.06)	0.014*
**Preoperative ECOG status**						
- ECOG ≤ 1	-		-		-	
- ECOG > 1	1.52 (1.04–2.22)	0.030*	1.88 (1.03–3.43)	0.038*	1.75 (0.94–3.25)	0.074
**Symptomatic BM**						
- No	-		-		-	
- Yes	0.98 (0.66–1.46)	> 0.90	0.72 (0.39–1.34)	0.30	0.69 (0.37–1.29)	0.25
**Number of BM**						
− 1	-		-		-	
− 2–5	1.54 (1.15–2.05)	0.003*	0.90 (0.53–1.52)	0.69	1.17 (0.72–1.89)	0.51
- >5	1.55 (1.00-2.39)	0.051	0.86 (0.38–1.93	0.71	1.13 (0.50–2.54)	0.75
**Extracranial metastases at BM surgery**						
- No	-		-		-	
- Yes	1.44 (1.10–1.88)	0.007*	1.93 (1.18–3.15)	0.008*	1.87 (1.15-3.05)	0.011*
**Steroid intake at hospital discharge**						
- No	-		-	-	-	-
- Yes	1.05 (0.75–1.47)	0.80	0.88 (0.46–1.66)	0.69	0.91 (0.47–1.76)	0.79
**Postoperative radiotherapy**						
- No	-					
- Yes	0.75 (0.51–1.11)	0.15				
**Postoperative SRT**						
- No	-					
- Yes	0.63 (0.47–0.83)	0.001*	-1.03 (0.59–1.80)	0.92	-1.06 (0.60–1.85)	0.83
**Postoperative WBRT**						
- No	-		-		-	
- Yes	1.44 (1.09–1.91)	0.011*	2.11 (1.20–3.69)	0.009*	2.23 (1.26–3.95)	0.005*
**Postoperative immunotherapy**						
- No	-		-		-	
- Yes	0.69 (0.50–0.94)	0.018*	0.57 (0.35–0.93)	0.024*	0.56 (0.34–0.92)	0.023*
**Postoperative TT**						
- No	-		-		-	
- Yes	0.73 (0.51–1.06)	0.095	0.59 (0.34–1.03)	0.064	0.62 (0.35–1.09)	0.10

BM, brain metastasis; ECOG, Eastern Cooperative Oncology Group; GTR, gross-total resection; SRT, stereotactic radiotherapy; TT, targeted therapy; WBRT, whole-brain radiotherapy

#Postoperative residual tumor volume values were log-transformed before fitting Cox regression model. *p value < 0.05

**Fig. 2:**
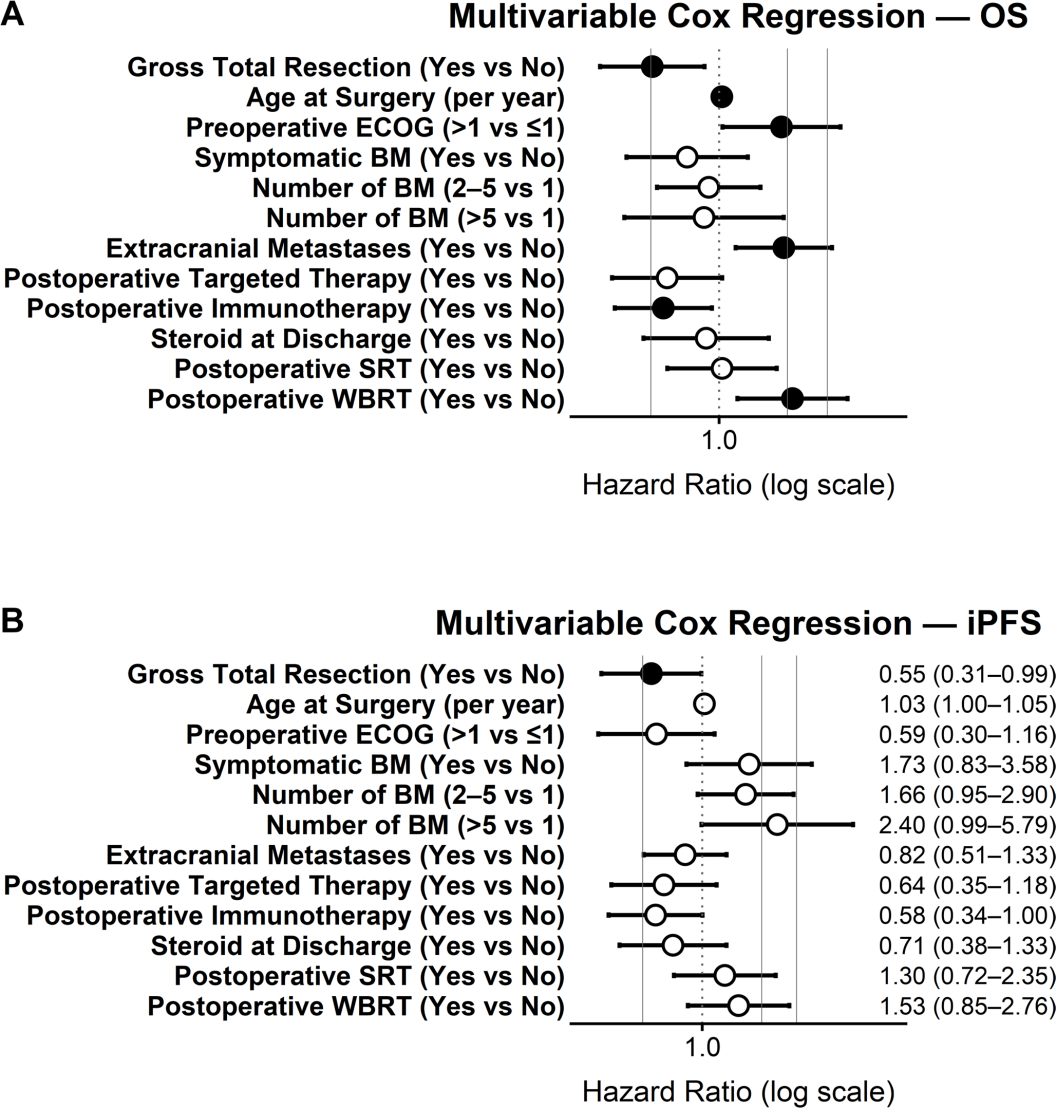
Multivariable cox regression analysis of overall survival and intracranial progression-free survival. Forest plots depicting adjusted hazard ratios (HRs) with 95% confidence intervals for predictors of (**A**) overall survival (OS) and (**B**) intracranial progression-free survival (iPFS) in the study cohort. Variables included in the multivariable Cox regression models were gross total resection, age at surgery, preoperative ECOG performance status, symptomatic brain metastasis (BM), number of brain metastases at surgery, presence of extracranial metastases, postoperative targeted therapy, postoperative immunotherapy, steroid at discharge, postoperative stereotactic radiotherapy (SRT), and postoperative whole-brain radiotherapy (WBRT). HRs are presented on a logarithmic scale, with values < 1 indicating association with reduced hazard and values > 1 indicating increased hazard

In multivariable Cox regression (Table 2; Fig. 2A), GTR remained independently associated with improved OS (adjusted HR 0.51; 95%CI, 0.30–0.86; *p* = 0.012), whereas postoperative RV was not significant (adjusted HR 1.14; 95%CI, 0.88–1.47; *p* = 0.31). Older age, ECOG > 1, the presence of extracranial metastases, and postoperative WBRT were independently associated with shorter OS, while postoperative immunotherapy – and to a lesser extent TT – was associated with improved OS.

Kaplan-Meier estimates showed superior survival in patients with GTR (median OS of 28.6 months; 95%CI, 17.9–42.7) compared to incomplete resection (12.2 months; 95%CI, 9.0-16.3, *p* < 0.001).

#### Stratified analysis by the number of BM at time of surgery

In patients with a single BM (*n* = 155), both GTR (adjusted HR 0.40; 95% CI, 0.21–0.76; *p* = 0 0.005) and lower postoperative RV (adjusted HR 1.43; 95% CI, 1.07–1.90; *p* = 0.012) were associated with longer OS, with median OS of 28.5 versus 15.7 months for GTR versus incomplete resection (*p* = 0.004; Fig. 1B). In contrast, in patients with multiple BM (*n* = 130), neither GTR nor postoperative RV showed an association with OS in either univariable or multivariable analysis. In this subgroup, postoperative TT was associated with improved OS, while ECOG > 1 and postoperative WBRT were associated with worse OS (Supplemental Tables 1, 2). The median OS was 15.1 months (95% CI, 9.46–24.7) with GTR, compared with 11.4 months (95% CI, 8.2–15.1; *p* = 0.14) with incomplete resection (Fig. 1B).

Additional subgroup analyses are provided in the Supplemental Materials (Supplemental Tables 3–8).

### Intracranial progression-free survival analysis

The median iPFS for the entire cohort was 8.0 months (95%CI, 6.3–10.6; Fig. 1D). In univariable analysis (Table 3), both GTR (HR 0.48; 95%CI, 0.34–0.67; *p* < 0.001) and lower postoperative RV (HR 1.20; 95%CI, 1.05–1.38; *p* = 0.009) were associated with longer iPFS.

**Table 3:** Cox regression analysis prognostic factors for progression-free survival

	Univariable		Multivariable			
	HR (95%CI)	*P* value	HR (95%CI)	*P* value	HR (95%CI)	*P* value
**Gross-total resection (GTR)**						
- No	-					
- Yes	0.48 (0.34–0.67)	< 0.001*	0.55 (0.31–0.99)	0.046*		
**Postoperative residual tumor volume [cm** ^**3**^ **]#**	1.20 (1.05–1.38)	0.009*			1.16 (0.90–1.50)	0.24
**Age at BM surgery**	1.00 (0.99–1.02)	0.70	1.03 (0.99–1.05)	0.057	1.03 (1.00-1.05)	0.024*
**Preoperative ECOG status**						
- ECOG ≤ 1	-		-		-	
- ECOG > 1	1.02 (0.62–1.67)	> 0.90	0.59 (0.30–1.16)	0.12	0.52 (0.25–1.10)	0.088
**Symptomatic BM**						
- No	-		-		-	
- Yes	1.34 (0.77–2.33)	0.30	1.73 (0.83–3.58)	0.14	1.54 (0.74–3.18)	0.23
**Number of BM**						
− 1	-		-		-	
− 2–5	1.96 (1.41–2.73)	**< 0.001**	1.66 (0.95–2.90)	0.076	2.09 (1.27–3.43)	0.003*
- >5	1.42 (0.85–2.37)	0.20	2.40 (0.99–5.79)	0.052	3.12 (1.33–7.27)	0.008*
**Extracranial metastases at BM surgery**						
- No	-		-		-	
- Yes	0.87 (0.64–1.18)	0.40	0.82 (0.51–1.33)	0.42	0.81 (0.50–1.31)	0.39
**Steroid intake at hospital discharge**						
- No	-		-	-	-	-
- Yes	0.85 (0.60–1.20)	0.30	0.71 (0.38–1.33)	0.28	0.74 (0.39–1.40)	0.36
**Postoperative radiotherapy**						
- No	-					
- Yes	1.36 (0.81–2.27)	0.20				
**Postoperative SRT**						
- No	-		-		-	
- Yes	1.11 (0.79–1.58)	0.50	1.30 (0.72–2.35)	0.38	1.36 (0.75–2.49)	0.30
**Postoperative WBRT**						
- No	-		-		-	
- Yes	1.22 (0.88–1.70)	0.20	1.53 (0.85–2.76)	0.16	1.64 (0.90-3.00)	0.10
**Postoperative immunotherapy**						
- No	-		-		-	
- Yes	0.84 (0.60–1.17)	0.30	0.58 (0.34-1.00)	0.051	0.56 (0.33–0.98)	0.36
**Postoperative TT**						
- No	-		-		-	
- Yes	0.89 (0.61–1.31)	0.60	0.64 (0.35–1.18)	0.15	0.68 (0.37–1.26)	0.23

BM, brain metastasis; ECOG, Eastern Cooperative Oncology Group; GTR, gross-total resection; SRT, stereotactic radiotherapy; TT, targeted therapy; WBRT, whole-brain radiotherapy

#Postoperative residual tumor volume values were log-transformed before fitting Cox regression model. *p value < 0.05

In multivariable analysis (Table 3; Fig. 2B), GTR remained independently associated with prolonged iPFS (adjusted HR 0.55; 95%CI, 0.31–0.99; *p* = 0.046), whereas postoperative RV was not (adjusted HR 1.16; 95%CI, 0.90–1.50; *p* = 0.24). Postoperative immunotherapy and steroid use showed trends toward improved iPFS; multiple BM and older age showed trends toward shorter iPFS.

Median iPFS was 13.1 months (95% CI, 9.3–18.0) after GTR versus 5.4 months (95% CI, 3.3–6.3; *p* < 0.001) after incomplete resection.

#### Stratified analysis by the number of BM at time of surgery

In patients with a single BM (*n* = 155), GTR was associated with longer iPFS in both univariable (HR 0.48; 95%CI, 0.30–0.77; *p* = 0.002) and multivariable analyses (adjusted HR 0.40; 95%CI, 0.19–0.84; *p* = 0.016), with median iPFS was 15.6 versus 5.6 months (*p* = 0.005; Fig. 1E). In patients with multiple BM (*n* = 130), GTR (adjusted HR 0.42; 95%CI, 0.15–1.16; *p* = 0.097) and lower postoperative RV (adjusted HR 1.43; 95%CI, 0.97–2.12; *p* = 0.066) showed nonsignificant trends toward longer iPFS, and steroid use at discharge was independently associated with improved iPFS (Supplemental Tables 9, 10). The median iPFS was 9.0 months (95% CI, 5.0-14.3) with GTR, compared with 4.5 months (95% CI, 2.7–6.6; *p* = 0.69) with incomplete resection (Fig. 1E).

Additional subgroup analyses are provided in the Supplemental Materials (Supplemental Tables 11–16).

### Postoperative residual volume and survival

Across all analyses, postoperative RV showed only limited and inconsistent association with OS and iPFS, whereas achieving GTR consistently correlated with improved survival outcomes. Restricted cubic spline analysis showed no dose–response relationship between RV and survival (Supplemental Fig. 4A**-**B). In multivariable analyses restricted to non-GTR patients, RV was not associated with OS or iPFS (Supplemental Table 17) and Cox analyses using predefined RV classes (0 cm^3^, > 0–1 cm^3^, > 1–5 cm^3^, > 5 cm^3^) showed the best OS and iPFS in the GTR group (Fig. 1C and F), while the three non-zero categories exhibited similar outcomes with overlapping confidence intervals and no dose-response pattern (0–1 cm^3^: adjusted HR 1.81, 95% CI 1.02–3.21; >1–5 cm^3^: adjusted HR 1.93, 95% CI 0.92–4.02; >5 cm^3^: adjusted HR 2.14, 95% CI 0.89–5.17).

## Discussion

### Main findings

In this two-center retrospective study of surgically treated NSCLC-BM patients, achieving complete intracranial tumor resection (GTR) consistently emerged as a strong predictor of improved OS and iPFS, whereas postoperative RV showed no dose-dependent prognostic value. Stratified subgroup analyses further underscored that the prognostic benefit of GTR was most evident in patients without extracranial metastases and those with a single brain lesion, and was observed regardless of postoperative immunotherapy use. Older age, the presence of extracranial metastases, and poor preoperative performance status were associated with worse OS, while the presence of multiple BM was independently associated with shorter iPFS.

### Postoperative intracranial tumor load reduction in relation to survival outcomes

In contrast to established prognostic models, such as Recursive Partitioning Analysis (RPA) [10] or Graded Prognostic Assessment (GPA) [2, 11], this study specifically evaluated the prognostic relevance of surgical tumor load reduction (measured as GTR and postoperative RV) within a multimodal oncological treatment framework.

Our findings show that GTR was consistently associated with longer OS and iPFS, in line with previous studies [12–20]. This benefit is biologically plausible, as complete macroscopic cytoreduction improves local disease control in BM, which often display a less infiltrative encapsulated growth pattern [21]. Local control is clinically relevant, as mortality in patients with lung cancer BM has been shown to be related to CNS progression [13]. Accordingly, the survival advantage of GTR in our cohort was most evident in patients without extracranial metastases (i.e., controlled systemic disease) and in those with a single metastasis (i.e., limited intracranial disease). In patients with multiple BM, only a small proportion (*n* = 11/130) achieved complete resection, limiting interpretability; nevertheless, even in this subgroup, Kaplan-Meier curves showed a non-significant trend toward improved outcomes with GTR. Further prospective studies are needed to clarify the role of maximal cytoreduction in selected patients with multifocal but resectable disease. Interestingly, GTR was associated with improved OS in both patients who did and did not receive postoperative immunotherapy, suggesting that maximal resection retains prognostic importance even in the era of effective systemic treatments such as ICI [22, 23].

While some prior studies have not showed a clear prognostic association of EOR in lung cancer patients with BM [24–26], such discrepancies likely reflect differences in study design and the intrinsic heterogeneity of NSCLC-BM – particularly variations in intracranial disease burden, systemic disease control, timing of BM diagnosis, and access to targeted or immune-based therapies. In such contexts, the prognostic value of GTR may be diluted by competing factors. To account for these intrinsic differences within the study population, we conducted extensive multivariable and subgroup analyses. However, despite these analyses, remaining confounding remains inherent to the retrospective design. Moreover, GTR may partially reflect favorable tumor biology and patient selection (e.g., solitary lesions, superficial location, good performance status), which independently correlate with improved outcomes. Prospective, stratified studies are therefore needed to better define which patients derive the greatest benefit from maximal resection.

In contrast, postoperative RV showed a weak, inconsistent, and non-dose-dependent association with OS and iPFS. In our cohort, prognostic separation was driven primarily by the presence of any measurable residual disease, not by the amount of RV. Several factors may explain this pattern. First, the presence of any postoperative RV likely represents a combination of unfavorable factors – such as multifocal intracranial disease, infiltrative tumor boundaries, eloquent-area involvement, or non-resectability due to proximity to critical structures. These factors may independently impair prognosis regardless of the absolute RV size, explaining why increasing RV did not demonstrate a clear dose-response relationship. Second, many non-GTR patients had small residual volumes (< 5 cm^3^), which may carry limited incremental clinical relevance, especially in the context of effective postoperative therapies such as stereotactic radiosurgery and systemic treatments. Finally, measurement variability due to postoperative changes, edema, or segmentation limitations may further weaken detectable associations between RV quantity and survival.

### Other variables affecting survival outcomes

The presence of extracranial metastases at surgery was a negative prognostic factor, consistent with existing evidence that systemic disease burden adversely affects OS in NSCLC-BM patients [27, 28]. Our findings underscore the emerging role of postoperative immunotherapy as an independent predictor of longer OS and showed a favorable trend for improved iPFS, reinforcing data that immune-based therapies substantially contribute to both systemic and intracranial disease control in NSCLC-BM [29, 30]. Interestingly, postoperative TT showed significance in univariable analyses for OS but did not retain its prognostic association in multivariable models. More specifically regarding iPFS, the presence of multiple BM consistently predicted earlier intracranial progression, aligning with prior observations that higher intracranial tumor burden compromises local control [28].

### Limitations

Although this study represents the largest analysis of surgically treated NSCLC brain metastases to date, several limitations should be acknowledged. Its retrospective design introduces potential selection bias and limits the assessment of intracranial progression, as follow-up MRI was not protocolized and progression was determined through multidisciplinary review. These data do not allow definitive conclusions regarding surgery versus other local therapies, such as stereotactic radiotherapy. Although the multidisciplinary treatment context enabled adjustment for major confounders, evolving systemic therapies over the study period may have influenced survival outcomes and modified the prognostic impact of GTR and residual volume. In addition, incomplete data on PD-L1 expression and molecular alterations necessitated the use of postoperative immunotherapy and targeted therapy as surrogates for tumor biology. Finally, cause of death was incompletely documented, limiting distinction between CNS- and extracranial disease–related mortality.

## Conclusion

In this two-center cohort of surgically treated NSCLC-BM patients managed within a multimodal oncological framework, achieving complete intracranial tumor resection was consistently associated with improved overall survival and intracranial disease control, whereas the extent of postoperative residual volume showed no dose-dependent prognostic value. These findings highlight the clinical importance of achieving GTR in selected patients (e.g., those with a single BM and no extracranial disease), support informed patient counselling and surgical decision-making, and warrant further investigation of the role of GTR in patients with multifocal but safely resectable intracranial disease.

## Supplementary Information

Below is the link to the electronic supplementary material.

Supplementary Material 1

## Data Availability

No datasets were generated or analysed during the current study.
