# Supplementary material for: Postoperative absence of residual intracranial tumor volume is associated with improved survival and intracranial disease control in non-small cell lung cancer brain metastases
Source: J Neurooncol. 2025 Dec 22;176(2):131. doi: 10.1007/s11060-025-05387-1 (PMC12722344; doi:10.1007/s11060-025-05387-1)
Supplement: Supplementary file 1 — Supplementary Material 1 [file 11060_2025_5387_MOESM1_ESM.docx]

## SUPPLEMENTAL RESULTS

**Supplemental results for OS analysis**

Stratified analysis by the presence of extracranial metastases at time of surgery

In patients with extracranial metastases (n = 137), postoperative RV and GTR were not associated with OS. The univariable Cox regression analysis showed that older age (HR 1.03; 95%CI, 1.01-1.05; p = 0.005) and ECOG performance status >1 (HR 2.05; 95%CI, 1.15-3.65; p = 0.015) were significantly associated with shorter OS. In contrast, postoperative immunotherapy (HR 0.57; 95%CI, 0.38-0.85; p = 0.006), postoperative TT (HR 0.62; 95%CI, 0.39-1.00; p = 0.050), and postoperative SRT (HR 0.61; 95%CI, 0.41-0.90; p = 0.013) were associated with improved OS. In multivariable models, ECOG status >1 emerged as the only independent predictor of worse OS.

In patients without extracranial metastases (n = 140), achievement of GTR (HR 0.37; 95%CI, 0.24-0.59; p <0.001) and lower postoperative RV (HR 1.18; 95%CI, 1.00-1.40; p = 0.049) associated with improved OS in univariable analysis. In contrast, older age (HR 1.04; 95%CI, 1.01-1.06; p = 0.001), a higher number of BM (2-5 lesions: HR 2.08; 95%CI, 1.37-3.17; p <0.001; > 5 lesions: HR 2.06; 95%CI, 0.93-4.57; p = 0.075), and postoperative WBRT (HR 1.58; 95%CI, 1.04-2.37; p = 0.030) were associated with poorer survival. In the multivariable model, GTR remained independently associated with improved OS (adjusted HR 0.42; 95%CI, 0.20-0.89; p = 0.024), whereas postoperative RV did not (**Supplemental Table 3**, **4**).

Stratified analysis by postoperative immunotherapy

In patients who received postoperative immunotherapy (n = 88), GTR was associated with improved OS in univariable and multivariable Cox regression models (HR 0.42; 95%CI, 0.24-0.76; p = 0.004; adjusted HR 0.43; 95%CI, 0.18-0.97; p = 0.043). Additionally, older age at the timepoint of surgery and postoperative WBRT was independently associated with shorter OS.

In patients who do not receive postoperative immunotherapy (n = 136), GTR (HR 0.48; 95%CI, 0.29-0.77; p = 0.003; adjusted HR 0.37; 95%CI, 0.18-0.78; p = 0.009) and lower postoperative RV (HR 1.21; 95%CI, 1.00-1.47; p = 0.050; adjusted HR 1.47; 95%CI, 1.04-2.08; p = 0.026) were independently associated with improved OS. The presence of extracranial metastases, preoperative ECOG status >1, and postoperative WBRT were associated with inferior survival. Postoperative SRT and TT were significantly associated with longer OS in univariable analysis; however, these associations did not remain significant in the multivariable model (**Supplemental Table 5**, **6**).

Stratified analysis by postoperative TT

In patients receiving postoperative TT (n = 46), none of the investigated variables – including postoperative RV or GTR – showed a consistent association with OS in Cox regression analyses.

In contrast, in patients not receiving postoperative TT (n = 177), GTR (HR 0.39; 95%CI, 0.26-0.59; p <0.001; adjusted HR 0.47; 95%CI, 0.25-0.85; p = 0.013) was significantly associated with improved OS in both univariable and multivariable models. Postoperative RV showed a significant association with OS only in the univariable analysis. In this subgroup, the presence of extracranial metastases remained an independent negative prognostic factor, while postoperative immunotherapy was independently associated with longer OS (**Supplemental Table 7**, **8**).

**Supplemental results for iPFS analysis**

Stratified analysis by the presence of extracranial metastases at time of surgery

In patients with extracranial metastases (n = 137), GTR achievement (HR 0.46; 95%CI, 0.26-0.81; p = 0.007; adjusted HR 0.25; 95%CI, 0.083-0.74; p = 0.013) was associated with longer iPFS both in univariable and multivariable models. However, no consistent association was found with postoperative RV or other investigated covariates.

In patients without extracranial metastases (n = 140), GTR was associated with improved iPFS (HR 0.45; 95%CI, 0.29-0.71; p <0.001) and the presence of multiple BM (2-5 lesions: HR 2.30; 95%CI, 1.48-3.58; p <0.001) with earlier intracranial progression in univariable analysis. In multivariable analysis, only the number of BM remained independently associated with shorter iPFS, while no association was found with GTR or postoperative RV (**Supplemental Table 9**, **10**).

Stratified analysis by postoperative immunotherapy

In patients receiving postoperative immunotherapy (n = 88), GTR was associated with longer iPFS in univariable analysis (HR 0.46; 95%CI, 0.26-0.83; p = 0.010), but the association did not remain significant in multivariable analysis. No consistent associations with iPFS were observed for RV or other variables in this subgroup.

In patients who did not receive postoperative immunotherapy (n = 136), GTR (HR 0.42, 95%CI 0.26-0.70, p <0.001; adjusted HR 0.45; 95%CI, 0.20-1.03; p = 0.060) and lower postoperative RV (HR 1.38, 95%CI 1.12-1.69, p = 0.002; adjusted HR 1.42; 95%CI, 0.99-2.04; p = 0.054) were associated with improved iPFS in univariable analysis and showed a trend toward significance in the multivariable model. Additionally, the presence of multiple BMs (2–5 lesions: HR 2.11; 95% CI, 1.34–3.33; p = 0.001) was associated with shorter iPFS in the univariable analysis; however, the association with did not remain significant in the multivariable model (**Supplemental Table 13**, **14**).

Stratified analysis by postoperative TT

In patients who received postoperative TT (n = 46), no consistent associations with iPFS were found for postoperative RV, GTR, or any other variables investigated.

In contrast, in patients not treated with postoperative TT (n = 177), GTR was significantly associated with prolonged iPFS (HR 0.37; 95%CI, 0.24-0.57; p <0.001; adjusted HR 0.39; 95%CI, 0.20-0.74; p = 0.004). Postoperative RV showed a significant association with iPFS only in the univariable analysis. In this subgroup, postoperative immunotherapy was independently associated with improved intracranial disease control (**Supplemental Table 15**, **16**).

## SUPPLEMENTAL TABLES AND FIGURES

|  | **Univariable Cox regression analysis (OS)** | | | |
| --- | --- | --- | --- | --- |
|  | **Single BM** | | **Multiple BM** | |
|  | HR (95%CI) | *P* value | HR (95%CI) | *P* value |
| **Gross-total resection (GTR)**  - No  - Yes | -  0.51 (0.33-0.77) | **0.002*** | -  0.49 (0.21-1.13) | 0.10 |
| **Postoperative residual tumor volume [cm^3^]#** | 1.26 (1.06-1.50) | **0.007*** | 1.01 (0.83-1.24) | >0.90 |
| **Age at BM surgery** | 1.04 (1.02-1.06) | **<0.001*** | 1.03 (1.01-1.05) | **0.011*** |
| **Preoperative ECOG status**  - ECOG ≤1  - ECOG >1 | -  1.62 (0.94-2.78) | 0.081 | -  1.48 (0.87-2.51) | 0.15 |
| **Symptomatic BM**  - No  - Yes | -  1.10 (0.65-1.86) | 0.70 | -  0.78 (0.42-1.42) | 0.40 |
| **Extracranial metastases**  - No  - Yes | -  1.80 (1.25-2.59) | **0.002*** | -  1.03 (0.69-1.53) | 0.90 |
| **Steroid intake at hospital discharge**  - No  - Yes | -  1.35 (0.83-2.18) | 0.20 | -  0.76 (0.47-1.22) | 0.30 |
| **Postoperative radiotherapy**  - No  - Yes | -  0.86 (0.49-1.50) | 0.60 | -  0.64 (0.38-1.10) | 0.11 |
| **Postoperative SRT**  - No  - Yes | -  0.61 (0.40-0.95) | **0.028*** | -  0.75 (0.50-1.11) | 0.20 |
| **Postoperative WBRT**  - No  - Yes | -  1.51 (0.99-2.30) | 0.054 | -  1.14 (0.77-1.69) | 0.50 |
| **Postoperative immunotherapy**  - No  - Yes | -  0.74 (0.49-1.14) | 0.20 | -  0.69 (0.43-1.09) | 0.11 |
| **Postoperative TT**  - No  - Yes | -  0.98 (0.60-1.58) | >0.90 | -  0.48 (0.27-0.85) | **0.012*** |

**Supplemental Table 1. Univariable Cox regression analysis for OS stratified by the number of intracranial lesions at timepoint of BM surgery.**

BM, brain metastasis; ECOG, Eastern Cooperative Oncology Group; GTR, gross-total resection; OS, overall survival; SRT, stereotactic radiotherapy; TT, targeted therapy; WBRT, whole-brain radiotherapy. #Postoperative residual tumor volume values were log-transformed before fitting Cox regression model. *p value < 0.05.

|  | **Multivariable Cox regression analysis (OS)** | | | | | | | |
| --- | --- | --- | --- | --- | --- | --- | --- | --- |
|  | **Single BM** | | | | **Multiple BM** | | | |
|  | HR (95%CI) | *P* value | HR (95%CI) | *P* value | HR (95%CI) | *P* value | HR (95%CI) | *P* value |
| **Gross-total resection (GTR)**  - No  - Yes | -  0.40 (0.21-0.76) | **0.005*** |  |  | -  0.48 (0.16-1.45) | 0.19 |  |  |
| **Postoperative residual tumor volume [cm^3^]#** |  |  | 1.43 (1.07-1.90) | **0.012*** |  |  | 1.01 (0.68-1.51) | 0.93 |
| **Age at BM surgery** | 1.04 (1.00-1.09) | **0.039*** | 1.04 (1.00-1.09) | **0.035*** | 1.03 (0.99-1.07) | 0.082 | 1.03 (0.99-1.07) | 0.056 |
| **Preoperative ECOG status**  - ECOG ≤1  - ECOG >1 | -  2.01 (0.78-5.19) | 0.14 | -  1.61 (0.59-4.41) | 0.34 | -  2.34 (1.07-5.10) | **0.032*** | -  2.29 (0.97-5.35) | 0.055 |
| **Symptomatic BM**  - No  - Yes | -  0.83 (0.34-2.02) | 0.69 | -  0.84 (0.35-2.04) | 0.71 | -  0.47 (0.19-1.18) | 0.11 | -  0.42 (0.17-1.07) | 0.072 |
| **Extracranial metastases**  - No  - Yes | -  2.54 (1.29-5.02) | **0.007*** | -  2.31 (1.20-4.44) | **0.012*** | -  1.48 (0.69-3.16) | 0.31 | -  1.53 (0.71-3.29) | 0.27 |
| **Steroid intake at hospital discharge**  - No  - Yes | -  1.88 (0.67-5.28) | 0.23 | -  2.25 (0.76-6.67) | 0.14 | -  0.31 (0.13-0.75) | **0.010*** | -  0.32 (0.13-0.77) | **0.011*** |
| **Postoperative SRT**  - No  - Yes | -  0.67 (0.29-1.58) | 0.36 | -  0.69 (0.30-1.62) | 0.40 | -  0.60 (0.30-1.20) | 0.15 | -  0.52 (0.26-1.05) | 0.070 |
| **Postoperative WBRT**  - No  - Yes | -  2.74 (1.12-6.70) | **0.026*** | -  2.64 (1.03-6.74) | **0.042*** | -  2.08 (1.07-4.06) | **0.030*** | -  2.30 (1.17-4.53) | **0.015*** |
| **Postoperative immunotherapy**  - No  - Yes | -  0.78 (0.38-1.59) | 0.49 | -  0.71 (0.34-1.49) | 0.37 | -  0.79 (0.37-1.71) | 0.56 | -  0.77 (0.36-1.67) | 0.52 |
| **Postoperative TT**  - No  - Yes | -  1.19 (0.54-2.63) | 0.66 | -  1.07 (0.47-2.43) | 0.85 | -  0.29 (0.13-0.69) | **0.004*** | -  0.31 (0.13-0.72) | **0.006*** |

**Supplemental Table 2. Multivariable Cox regression analysis for OS stratified by the presence of extracranial metastases at timepoint of BM surgery.**

BM, brain metastasis; ECOG, Eastern Cooperative Oncology Group; GTR, gross-total resection; OS, overall survival; SRT, stereotactic radiotherapy; TT, targeted therapy; WBRT, whole-brain radiotherapy. #Postoperative residual tumor volume values were log-transformed before fitting Cox regression model. *p value < 0.05.

|  | **Univariable Cox regression analysis (OS)** | | | |
| --- | --- | --- | --- | --- |
|  | **Extracranial disease positive status** | | **Extracranial disease negative status** | |
|  | HR (95%CI) | *P* value | HR (95%CI) | *P* value |
| **Gross-total resection (GTR)**  - No  - Yes | -  0.66 (0.42-1.06) | 0.086 | -  0.37 (0.24-0.59) | **<0.001*** |
| **Postoperative residual tumor volume [cm^3^]#** | 1.17 (0.95-1.44) | 0.13 | 1.18 (1.00-1.40) | **0.049*** |
| **Age at BM surgery** | 1.03 (1.01-1.05) | **0.005*** | 1.04 (1.01-1.06) | **0.001*** |
| **Preoperative ECOG status**  - ECOG ≤1  - ECOG >1 | -  2.05 (1.15-3.65) | **0.015*** | -  1.50 (0.88-2.54) | 0.13 |
| **Symptomatic BM**  - No  - Yes | -  0.83 (0.49-1.38) | 0.50 | -  1.16 (0.62-2.17) | 0.60 |
| **Number of BM**  - 1  - 2-5  - >5 | -  1.12 (0.74-1.68)  1.03 (0.59-1.80) | 0.60  >0.90 | -  2.08 (1.37-3.17)  2.06 (0.93-4.57) | **<0.001***  0.0075 |
| **Steroid intake at hospital discharge**  - No  - Yes | -  1.00 (0.59-1.70) | >0.90 | -  1.12 (0.70-1.79) | 0.60 |
| **Postoperative radiotherapy**  - No  - Yes | -  0.73 (0.44-1.21) | 0.20 | -  0.88 (0.47-1.64) | 0.70 |
| **Postoperative SRT**  - No  - Yes | -  0.61 (0.41-0.90) | **0.013*** | -  0.76 (0.49-1.18) | 0.20 |
| **Postoperative WBRT**  - No  - Yes | -  1.20 (0.80-1.79) | 0.40 | -  1.58 (1.04-2.37) | **0.030*** |
| **Postoperative immunotherapy**  - No  - Yes | -  0.57 (0.38-0.85) | **0.006*** | -  0.76 (0.46-1.27) | 0.30 |
| **Postoperative TT**  - No  - Yes | -  0.62 (0.39-1.00) | 0.050 | -  0.82 (0.45-1.50) | 0.50 |

**Supplemental Table 3. Univariable Cox regression analysis for OS stratified by the presence of extracranial metastases at timepoint of BM surgery.**

BM, brain metastasis; ECOG, Eastern Cooperative Oncology Group; GTR, gross-total resection; OS, overall survival; SRT, stereotactic radiotherapy; TT, targeted therapy; WBRT, whole-brain radiotherapy. #Postoperative residual tumor volume values were log-transformed before fitting Cox regression model. *p value < 0.05.

|  | **Multivariable Cox regression analysis (OS)** | | | | | | | |
| --- | --- | --- | --- | --- | --- | --- | --- | --- |
|  | **Extracranial disease positive status** | | | | **Extracranial disease negative status** | | | |
|  | HR (95%CI) | *P* value | HR (95%CI) | *P* value | HR (95%CI) | *P* value | HR (95%CI) | *P* value |
| **Gross-total resection (GTR)**  - No  - Yes | -  0.52 (0.22-1.19) | 0.12 |  |  | -  0.42 (0.20-0.89) | **0.024*** |  |  |
| **Postoperative residual tumor volume [cm^3^]#** |  |  | 1.11 (0.67-1.82) | 0.67 |  |  | 1.24 (0.87-1.76) | 0.23 |
| **Age at BM surgery** | 1.04 (0.99-1.08) | 0.069 | 1.03 (0.99-1.07) | 0.085 | 1.03 (0.99-1.07) | 0.065 | 1.05 (1.01-1.09) | **0.011*** |
| **Preoperative ECOG status**  - ECOG ≤1  - ECOG >1 | -  20.38 (4.98-83.37) | **<0.001*** | -  22.27 (5.17-95.89) | **<0.001*** | -  1.01 (0.48-2.12) | 0.96 | -  0.90 (0.42-1.93) | 0.79 |
| **Symptomatic BM**  - No  - Yes | -  0.83 (0.38-1.81) | 0.64 | -  0.89 (0.41-1.93) | 0.78 | -  1.43 (0.50-4.07) | 0.49 | -  1.13 (0.41-3.10) | 0.80 |
| **Number of BM**  - 1  - 2-5  - >5 | -  0.67 (0.28-1.57)  0.62 (0.20-1.88) | 0.36  0.40 | -  0.94 (0.45-1.98)  0.78 (0.24-2.57) | 0.88  0.69 | -  1.23 (0.58-2.62)  1.31 (0.33-5.13) | 0.57  0.68 | -  1.54 (0.73-3.26)  1.79 (0.43-7.41) | 0.25  0.41 |
| **Steroid intake at hospital discharge**  - No  - Yes | -  0.89 (0.30-2.66) | 0.83 | -  0.90 (0.29-2.71) | 0.85 | -  0.77 (0.31-1.87) | 0.56 | -  0.85 (0.35-2.08) | 0.73 |
| **Postoperative SRT**  - No  - Yes | -  0.78 (0.37-1.65) | 0.51 | -  0.79 (0.37-1.69) | 0.55 | -  0.66 (0.28-1.53) | 0.33 | -  0.61 (0.26-1.43) | 0.25 |
| **Postoperative WBRT**  - No  - Yes | -  1.47 (0.69-3.18) | 0.32 | -  1.51 (0.70-3.25) | 0.29 | -  4.01 (1.72-9.40) | **0.001*** | -  4.32 (1.87-9.97) | **<0.001*** |
| **Postoperative immunotherapy**  - No  - Yes | -  0.91 (0.39-2.09) | 0.51 | -  0.86 (0.36-2.02) | 0.73 | -  0.80 (0.37-1.74) | 0.58 | -  0.74 (0.33-1.62) | 0.45 |
| **Postoperative TT**  - No  - Yes | -  0.66 (0.28-1.56) | 0.35 | -  0.72 (0.30-1.70) | 0.45 | -  0.73 (0.31-1.73) | 0.48 | -  0.69 (0.28-1.67) | 0.41 |

**Supplemental Table 4. Multivariable Cox regression analysis for OS stratified by the presence of extracranial metastases at timepoint of BM surgery.**

BM, brain metastasis; ECOG, Eastern Cooperative Oncology Group; GTR, gross-total resection; OS, overall survival; SRT, stereotactic radiotherapy; TT, targeted therapy; WBRT, whole-brain radiotherapy. #Postoperative residual tumor volume values were log-transformed before fitting Cox regression model. *p value < 0.05.

|  | **Univariable Cox regression analysis (OS)** | | | |
| --- | --- | --- | --- | --- |
|  | **Postoperative immunotherapy** | | **No postoperative immunotherapy** | |
|  | HR (95%CI) | *P* value | HR (95%CI) | *P* value |
| **Gross-total resection (GTR)**  - No  - Yes | -  0.42 (0.24-0.76) | **0.004*** | -  0.48 (0.29-0.77) | **0.003*** |
| **Postoperative residual tumor volume [cm^3^]#** | 1.24 (0.96-1.60) | 0.095 | 1.21 (1.00-1.47) | 0.050 |
| **Age at BM surgery** | 1.03 (1.00-1.06) | **0.041*** | 1.02 (1.00-1.04) | **0.033*** |
| **Preoperative ECOG status**  - ECOG ≤1  - ECOG >1 | -  1.19 (0.42-3.33) | 0.70 | -  1.88 (1.12-3.13) | **0.016*** |
| **Symptomatic BM**  - No  - Yes | -  1.12 (0.58-2.15) | 0.70 | -  0.78 (0.44-1.39) | 0.40 |
| **Number of BM**  - 1  - 2-5  - >5 | -  1.20 (0.66-2.16)  3.81 (1.81-8.01) | 0.50  **<0.001*** | -  1.66 (1.12-2.47)  1.11 (0.56-2.19) | **0.012***  0.80 |
| **Extracranial metastases**  - No  - Yes | -  1.34 (0.79-2.27) | 0.30 | -  1.73 (1.18-2.53) | **0.005*** |
| **Steroid intake at hospital discharge**  - No  - Yes | -  1.03 (0.49-2.19) | >0.90 | -  1.03 (0.66-1.59) | >0.90 |
| **Postoperative radiotherapy**  - No  - Yes | -  2.66 (0.83-8.56) | 0.10 | -  0.51 (0.32-0.83) | **0.006*** |
| **Postoperative SRT**  - No  - Yes | -  0.99 (0.54-1.83) | >0.90 | -  0.53 (0.36-0.77) | **<0.001*** |
| **Postoperative WBRT**  - No  - Yes | -  2.15 (1.27-3.64) | **0.005*** | -  1.36 (0.92-2.02) | 0.13 |
| **Postoperative TT**  - No  - Yes | -  0.89 (0.45-1.76) | 0.70 | -  0.60 (0.39-0.93) | **0.024*** |

**Supplemental Table 5. Univariable Cox regression analysis for OS stratified by the administration of postoperative immunotherapy.**

BM, brain metastasis; ECOG, Eastern Cooperative Oncology Group; GTR, gross-total resection; OS, overall survival; SRT, stereotactic radiotherapy; TT, targeted therapy; WBRT, whole-brain radiotherapy. #Postoperative residual tumor volume values were log-transformed before fitting Cox regression model. *p value < 0.05.

|  | **Multivariable Cox regression analysis (OS)** | | | | | | | |
| --- | --- | --- | --- | --- | --- | --- | --- | --- |
|  | **Postoperative immunotherapy** | | | | **No postoperative immunotherapy** | | | |
|  | HR (95%CI) | *P* value | HR (95%CI) | *P* value | HR (95%CI) | *P* value | HR (95%CI) | *P* value |
| **Gross-total resection (GTR)**  - No  - Yes | -  0.42 (0.18-0.97) | **0.043*** |  |  | -  0.37 (0.18-0.78) | **0.009*** |  |  |
| **Postoperative residual tumor volume [cm^3^]#** |  |  | 1.09 (0.65-1.81) | 0.73 |  |  | 1.47 (1.04-2.08) | **0.026*** |
| **Age at BM surgery** | 1.07 (1.02-1.12) | **0.002*** | 1.06 (1.02-1.11) | **0.004*** | 1.01 (0.98-1.04) | 0.48 | 1.01 (0.98-1.05) | 0.21 |
| **Preoperative ECOG status**  - ECOG ≤1  - ECOG >1 | -  1.06 (0.31-3.59) | 0.91 | -  1.23 (0.34-4.37) | 0.74 | -  3.02 (1.48-6.17) | **0.002*** | -  2.27 (1.10-4.69) | **0.025*** |
| **Symptomatic BM**  - No  - Yes | -  1.14 (0.42-3.06) | 0.79 | -  1.06 (0.41-2.72) | 0.90 | -  0.96 (0.40-2.30) | 0.93 | -  1.08 (0.42-2.77) | 0.85 |
| **Number of BM**  - 1  - 2-5  - >5 | -  0.75 (0.30-1.88)  5.02 (1.52-16.51) | 0.54  **0.008*** | -  1.13 (0.48-2.67)  7.67 (2.12-27.81) | 0.77  **0.001*** | -  1.01 (0.52-1.97)  0.30 (0.09-1.01) | 0.95  0.053 | -  1.25 (0.67-2.36)  0.33 (0.09-1.18) | 0.47  0.090 |
| **Extracranial metastases**  - No  - Yes | -  1.60 (0.70-3.65) | 0.26 | -  1.55 (0.67-3.55) | 0.29 | -  2.23 (1.14-4.35) | **0.018*** | -  2.43 (1.21-4.86) | **0.011*** |
| **Steroid intake at hospital discharge**  - No  - Yes | -  1.66 (0.51-5.44) | 0.40 | -  1.44 (0.45-4.61) | 0.53 | -  0.79 (0.31-2.01) | 0.62 | -  0.92 (0.35-2.39) | 0.87 |
| **Postoperative SRT**  - No  - Yes | -  1.49 (0.61-3.63) | 0.37 | -  1.29 (0.52-3.17) | 0.57 | -  0.55 (0.28-1.10) | 0.096 | -  0.56 (0.29-1.11) | 0.098 |
| **Postoperative WBRT**  - No  - Yes | -  1.94 (0.23-4.19) | 0.092 | -  2.17 (1.01-4.66) | **0.045*** | -  2.29 (1.16-4.52) | **0.017*** | -  2.28 (1.14-4.56) | **0.019*** |
| **Postoperative TT**  - No  - Yes | -  0.81 (0.28-2.89) | 0.75 | -  1.01 (0.29-3.44) | 0.98 | -  0.58 (0.30-1.11) | 0.10 | -  0.53 (0.27-1.05) | 0.069 |

**Supplemental Table 6. Multivariable Cox regression analysis for OS stratified by the administration of postoperative immunotherapy.**

BM, brain metastasis; ECOG, Eastern Cooperative Oncology Group; GTR, gross-total resection; OS, overall survival; SRT, stereotactic radiotherapy; TT, targeted therapy; WBRT, whole-brain radiotherapy. #Postoperative residual tumor volume values were log-transformed before fitting Cox regression model. *p value < 0.05.

|  | **Univariable Cox regression analysis (OS)** | | | |
| --- | --- | --- | --- | --- |
|  | **Postoperative TT** | | **No postoperative TT** | |
|  | HR (95%CI) | *P* value | HR (95%CI) | *P* value |
| **Gross-total resection (GTR)**  - No  - Yes | -  0.69 (0.29-1.63) | 0.40 | -  0.39 (0.26-0.59) | **<0.001*** |
| **Postoperative residual tumor volume [cm^3^]#** | 1.07 (0.72-1.58) | 0.70 | 1.27 (1.08-1.50) | **0.004*** |
| **Age at BM surgery** | 1.03 (1.00-1.06) | 0.10 | 1.03 (1.00-1.05) | **0.024*** |
| **Preoperative ECOG status**  - ECOG ≤1  - ECOG >1 | -  2.64 (0.75-9.26) | 0.13 | -  1.67 (1.03-2.71) | **0.038*** |
| **Symptomatic BM**  - No  - Yes | -  0.65 (0.27-1.59) | 0.30 | -  1.04 (0.63-1.71) | 0.90 |
| **Number of BM**  - 1  - 2-5  - >5 | -  1.24 (0.57-2.71)  0.91 (0.31-2.70) | 0.60  0.90 | -  1.75 (1.22-2.52)  2.25 (1.29-3.91) | **0.003***  **0.004*** |
| **Extracranial metastases**  - No  - Yes | -  1.28 (0.64-2.56) | 0.50 | -  1.58 (1.12-2.22) | **0.009*** |
| **Steroid intake at hospital discharge**  - No  - Yes | -  0.38 (0.16-0.92) | **0.031*** | -  1.16 (0.76-1.76) | 0.50 |
| **Postoperative radiotherapy**  - No  - Yes | -  0.77 (0.29-2.02) | 0.60 | -  0.63 (0.39-1.01) | 0.055 |
| **Postoperative SRT**  - No  - Yes | -  0.65 (0.32-1.29) | 0.20 | -  0.51 (0.36-0.73) | **<0.001*** |
| **Postoperative WBRT**  - No  - Yes | -  1.50 (0.74-3.05) | 0.30 | -  1.58 (1.04-2.37) | **0.030*** |
| **Postoperative immunotherapy**  - No  - Yes | -  0.80 (0.38-1.68) | 0.60 | -  0.59 (0.42-0.84 ) | **0.003*** |

**Supplemental Table 7. Univariable Cox regression analysis for OS stratified by the administration of postoperative TT.**

BM, brain metastasis; ECOG, Eastern Cooperative Oncology Group; GTR, gross-total resection; OS, overall survival; SRT, stereotactic radiotherapy; TT, targeted therapy; WBRT, whole-brain radiotherapy. #Postoperative residual tumor volume values were log-transformed before fitting Cox regression model. *p value < 0.05.

|  | **Multivariable Cox regression analysis (OS)** | | | | | | | |
| --- | --- | --- | --- | --- | --- | --- | --- | --- |
|  | **Postoperative TT** | | | | **No postoperative TT** | | | |
|  | HR (95%CI) | *P* value | HR (95%CI) | *P* value | HR (95%CI) | *P* value | HR (95%CI) | *P* value |
| **Gross-total resection (GTR)**  - No  - Yes | -  0.91 (0.21-4.01) | 0.91 |  |  | -  0.47 (0.25-0.85) | **0.013*** |  |  |
| **Postoperative residual tumor volume [cm^3^]#** |  |  | 1.64 (0.69-3.93) | 0.26 |  |  | 1.12 (0.81-1.53) | 0.47 |
| **Age at BM surgery** | 1.05 (0.99-1.12) | 0.079 | 1.07 (1.00-1.14) | **0.036*** | 1.02 (0.99-1.05) | 0.13 | 1.03 (0.99-1.06) | 0.053 |
| **Preoperative ECOG status**  - ECOG ≤1  - ECOG >1 | -  6.36 (1.05-38.36) | **0.043*** | -  4.24 (0.63-28.18) | 0.13 | -  1.87 (0.96-3.62) | 0.063 | -  1.68 (0.85-3.33) | 0.13 |
| **Symptomatic BM**  - No  - Yes | -  0.63 (0.14-2.71) | 0.53 | -  0.76 (0.17-3.35) | 0.71 | -  0.77 (0.36-1.63) | 0.50 | -  0.81 (0.38-1.71) | 0.58 |
| **Number of BM**  - 1  - 2-5  - >5 | -  0.74 (0.19-2.94)  0.36 (0.063-2.15) | 0.67  0.26 | -  0.60 (0.16-2.18)  0.27 (0.04-1.64) | 0.44  0.15 | -  1.03 (0.57-1.87)  1.57 (0.62-3.96) | 0.36  0.33 | -  1.40 (0.80-2.43)  2.23 (0.84-5.87) | 0.23  0.10 |
| **Extracranial metastases**  - No  - Yes | -  1.37 (0.40-4.62) | 0.60 | -  1.93 (0.49-7.54) | 0.34 | -  2.19 (1.23-3.91) | **0.007*** | -  2.08 (1.18-3.68) | **0.011*** |
| **Steroid intake at hospital discharge**  - No  - Yes | -  0.21 (0.038-1.23) | 0.084 | -  0.29 (0.05-1.71) | 0.17 | -  0.93 (0.43-1.99) | 0.86 | -  1.01 (0.47-2.16) | 0.97 |
| **Postoperative SRT**  - No  - Yes | -  0.53 (0.15-1.80) | 0.31 | -  0.49 (0.14-1.67) | 0.25 | -  0.85 (0.46-1.55) | 0.60 | -  0.83 (0.45-1.52) | 0.54 |
| **Postoperative WBRT**  - No  - Yes | -  3.64 (0.58-23.01) | 0.17 | -  7.59 (0.99-58.05) | 0.050 | -  2.00 (1.12-3.57) | **0.018*** | -  2.11 (1.19-3.76) | **0.010*** |
| **Postoperative immunotherapy**  - No  - Yes | -  1.08 (0.24-4.97) | 0.91 | -  1.14 (0.23-5.51) | 0.86 | -  0.56 (0.32-0.95) | **0.034*** | -  0.55 (0.32-0.94) | **0.028*** |

**Supplemental Table 8. Multivariable Cox regression analysis for OS stratified by the administration of postoperative TT.**

BM, brain metastasis; ECOG, Eastern Cooperative Oncology Group; GTR, gross-total resection; OS, overall survival; SRT, stereotactic radiotherapy; TT, targeted therapy; WBRT, whole-brain radiotherapy. #Postoperative residual tumor volume values were log-transformed before fitting Cox regression model. *p value < 0.05.

|  | **Univariable Cox regression analysis (iPFS)** | | | |
| --- | --- | --- | --- | --- |
|  | **Single BM** | | **Multiple BM** | |
|  | HR (95%CI) | *P* value | HR (95%CI) | *P* value |
| **Gross-total resection (GTR)**  - No  - Yes | -  0.48 (0.30-0.77) | **0.002*** | -  0.80 (0.37-1.76) | 0.60 |
| **Postoperative residual tumor volume [cm^3^]#** | 1.14 (0.95-1.36) | 0.20 | 1.03 (0.82-1.28) | 0.80 |
| **Age at BM surgery** | 1.02 (0.99-1.04) | 0.14 | 1.00 (0.97-1.02) | 0.60 |
| **Preoperative ECOG status**  - ECOG ≤1  - ECOG >1 | -  1.31 (0.68-2.53) | 0.40 | -  0.82 (0.39-1.74) | 0.60 |
| **Symptomatic BM**  - No  - Yes | -  1.59 (0.73-3.49) | 0.20 | -  1.07 (0.49-2.32) | 0.90 |
| **Extracranial metastases**  - No  - Yes | -  0.93 (0.61-1.41) | 0.70 | -  0.68 (0.44-1.07) | 0.10 |
| **Steroid intake at hospital discharge**  - No  - Yes | -  0.87 (0.54-1.40) | 0.60 | -  0.72 (0.43-1.21) | 0.20 |
| **Postoperative radiotherapy**  - No  - Yes | -  2.47 (1.00-6.10) | 0.050 | -  0.87 (0.46-1.65) | 0.70 |
| **Postoperative SRT**  - No  - Yes | -  1.59 (0.86-2.92) | 0.14 | -  1.25 (0.79-1.97) | 0.30 |
| **Postoperative WBRT**  - No  - Yes | -  1.47 (0.89-2.42) | 0.13 | -  0.76 (0.49-1.19) | 0.20 |
| **Postoperative immunotherapy**  - No  - Yes | -  0.86 (0.54-1.36) | 0.50 | -  0.81 (0.50-1.31) | 0.40 |
| **Postoperative TT**  - No  - Yes | -  0.91 (0.53-1.56) | 0.70 | -  0.83 (0.48-1.43) | 0.50 |

**Supplemental Table 9. Univariable Cox regression analysis for iPFS stratified by the number of intracranial lesions at timepoint of BM surgery.**

BM, brain metastasis; ECOG, Eastern Cooperative Oncology Group; GTR, gross-total resection; iPFS, intracranial progression-free survival; SRT, stereotactic radiotherapy; TT, targeted therapy; WBRT, whole-brain radiotherapy. #Postoperative residual tumor volume values were log-transformed before fitting Cox regression model. *p value < 0.05.

|  | **Multivariable Cox regression analysis (iPFS)** | | | | | | | |
| --- | --- | --- | --- | --- | --- | --- | --- | --- |
|  | **Single BM** | | | | **Multiple BM** | | | |
|  | HR (95%CI) | *P* value | HR (95%CI) | *P* value | HR (95%CI) | *P* value | HR (95%CI) | *P* value |
| **Gross-total resection (GTR)**  - No  - Yes | -  0.40 (0.19-0.84) | **0.016*** |  |  | -  0.42 (0.15-1.16) | 0.097 |  |  |
| **Postoperative residual tumor volume [cm^3^]#** |  |  | 1.14 (0.86-1.51) | 0.34 |  |  | 1.43 (0.97-2.12) | 0.066 |
| **Age at BM surgery** | 1.02 (0.98-1.07) | 0.23 | 1.02 (0.97-1.06) | 0.33 | 1.02 (0.99-1.06) | 0.084 | 1.04 (1.00-1.07) | **0.012*** |
| **Preoperative ECOG status**  - ECOG ≤1  - ECOG >1 | -  0.79 (0.78-5.19) | 0.14 | -  0.71 (0.25-2.01) | 0.52 | -  0.57 (0.21-1.56) | 0.28 | -  0.39 (0.12-1.22) | 0.10 |
| **Symptomatic BM**  - No  - Yes | -  0.83 (0.30-2.10) | 0.64 | -  0.96 (0.28-3.23) | 0.95 | -  1.94 (0.72-5.23) | 0.18 | -  2.01 (0.71-5.69) | 0.18 |
| **Extracranial metastases**  - No  - Yes | -  0.90 (0.42-1.90) | 0.79 | -  0.82 (0.39-1.72) | 0.53 | -  0.66 (0.32-1.35) | 0.26 | -  0.64 (0.31-1.33) | 0.23 |
| **Steroid intake at hospital discharge**  - No  - Yes | -  1.88 (0.67-5.28) | 0.23 | -  1.64 (0.58-4.58) | 0.34 | -  0.30 (0.13-0.72) | **<0.001*** | -  0.35 (0.15-0.81) | **0.014*** |
| **Postoperative SRT**  - No  - Yes | -  1.13 (0.40-3.21) | 0.81 | -  1.30 (0.46-3.64) | 0.61 | -  1.08 (0.52-2.24) | 0.83 | -  1.18 (0.54-2.56) | 0.67 |
| **Postoperative WBRT**  - No  - Yes | -  1.38 (0.44-4.26) | 0.57 | -  1.76 (0.57-5.43) | 0.32 | -  1.45 (0.72-2.90) | 0.28 | -  1.40 (0.67-2.91) | 0.36 |
| **Postoperative immunotherapy**  - No  - Yes | -  0.62 (0.29-1.33) | 0.22 | -  0.58 (0.26-1.27) | 0.17 | -  0.77 (0.34-1.76) | 0.54 | -  0.69 (0.30-1.62) | 0.40 |
| **Postoperative TT**  - No  - Yes | -  0.72 (0.28-1.82) | 0.49 | -  0.80 (0.30-2.11) | 0.17 | -  0.58 (0.24-1.36) | 0.21 | -  0.57 (0.24-1.37) | 0.21 |

**Supplemental Table 10. Multivariable Cox regression analysis for iPFS stratified by the number of intracranial lesions at timepoint of BM surgery.**

BM, brain metastasis; ECOG, Eastern Cooperative Oncology Group; GTR, gross-total resection; iPFS, intracranial progression-free survival; SRT, stereotactic radiotherapy; TT, targeted therapy; WBRT, whole-brain radiotherapy. #Postoperative residual tumor volume values were log-transformed before fitting Cox regression model. *p value < 0.05.

|  | **Univariable Cox regression analysis (iPFS)** | | | |
| --- | --- | --- | --- | --- |
|  | **Extracranial disease positive status** | | **Extracranial disease negative status** | |
|  | HR (95%CI) | *P* value | HR (95%CI) | *P* value |
| **Gross-total resection (GTR)**  - No  - Yes | -  0.46 (0.26-0.81) | **0.007*** | -  0.45 (0.29-0.71) | **<0.001*** |
| **Postoperative residual tumor volume [cm^3^]#** | 1.19 (0.97-1.46) | 0.10 | 1.24 (1.03-1.51) | **0.025*** |
| **Age at BM surgery** | 1.00 (0.97-1.02) | 0.80 | 1.01 (0.98-1.03) | 0.50 |
| **Preoperative ECOG status**  - ECOG ≤1  - ECOG >1 | -  1.03 (0.41-2.61) | >0.90 | -  0.83 (0.45-1.53) | 0.60 |
| **Symptomatic BM**  - No  - Yes | -  1.42 (0.65-3.10) | 0.40 | -  1.13 (0.52-2.46) | 0.80 |
| **Number of BM**  - 1  - 2-5  - >5 | -  1.60 (0.96-2.66)  1.51 (0.81-2.81) | 0.073  0.20 | -  2.30 (1.48-3.58)  1.11 (0.35-3.60) | **<0.001***  0.90 |
| **Steroid intake at hospital discharge**  - No  - Yes | -  0.72 (0.43-1.23) | 0.20 | -  1.06 (0.64-1.73) | 0.80 |
| **Postoperative radiotherapy**  - No  - Yes | -  1.64 (0.79-3.42) | 0.20 | -  1.14 (0.52-2.48) | 0.80 |
| **Postoperative SRT**  - No  - Yes | -  1.06 (0.65-1.72) | 0.80 | -  1.26 (0.74-2.17) | 0.40 |
| **Postoperative WBRT**  - No  - Yes | -  1.53 (0.94-2.50) | 0.085 | -  0.99 (0.62-1.59) | >0.90 |
| **Postoperative immunotherapy**  - No  - Yes | -  1.04 (0.65-1.68) | 0.90 | -  0.74 (0.45-1.24) | 0.30 |
| **Postoperative TT**  - No  - Yes | -  0.75 (0.45-1.27) | 0.30 | -  1.34 (0.74-2.40) | 0.30 |

**Supplemental Table 11. Univariable Cox regression analysis for iPFS stratified by the presence of extracranial metastases at timepoint of BM surgery.**

BM, brain metastasis; ECOG, Eastern Cooperative Oncology Group; GTR, gross-total resection; iPFS, intracranial progression-free survival; SRT, stereotactic radiotherapy; TT, targeted therapy; WBRT, whole-brain radiotherapy. #Postoperative residual tumor volume values were log-transformed before fitting Cox regression model. *p value < 0.05.

|  | **Multivariable Cox regression analysis (iPFS)** | | | | | | | |
| --- | --- | --- | --- | --- | --- | --- | --- | --- |
|  | **Extracranial disease positive status** | | | | **Extracranial disease negative status** | | | |
|  | HR (95%CI) | *P* value | HR (95%CI) | *P* value | HR (95%CI) | *P* value | HR (95%CI) | *P* value |
| **Gross-total resection (GTR)**  - No  - Yes | -  0.25 (0.083-0.74) | **0.013*** |  |  | -  0.58 (0.25-1.31) | 0.19 |  |  |
| **Postoperative residual tumor volume [cm^3^]#** |  |  | 1.13 (0.70-1.83) | 0.60 |  |  | 1.32 (0.92-1.88) | 0.12 |
| **Age at BM surgery** | 1.06 (1.01-1.11) | **0.009*** | 1.04 (0.99-1.09) | 0.059 | 1.01 (0.97-1.05) | 0.38 | 1.02 (0.99-1.06) | 0.12 |
| **Preoperative ECOG status**  - ECOG ≤1  - ECOG >1 | -  1.17 (0.19-6.92) | 0.86 | -  1.37 (0.22-8.48) | 0.72 | -  0.50 (0.22-1.13) | 0.098 | -  0.38 (0.14-0.99) | **0.048*** |
| **Symptomatic BM**  - No  - Yes | -  1.46 (0.54-3.96) | 0.45 | -  1.81 (0.67-4.84) | 0.23 | -  2.49 (0.72-8.64) | 0.14 | -  2.12 (0.66-6.79) | 0.20 |
| **Number of BM**  - 1  - 2-5  - >5 | -  0.71 (0.28-1.86)  1.72 (0.55-5.36) | 0.49  0.34 | -  1.53 (0.68-3.46)  2.81 (0.80-9.84) | 0.29  0.10 | -  2.13 (1.00-4.53)  2.57 (0.50-13.28) | **0.047***  0.25 | -  2.52 (1.21-5.24)  3.04 (0.66-13.95) | **0.012***  0.15 |
| **Steroid intake at hospital discharge**  - No  - Yes | -  0.44 (0.15-1.28) | 0.13 | -  0.40 (0.13-1.17) | 0.094 | -  0.84 (0.33-2.12) | 0.72 | -  0.92 (0.36-2.35) | 0.87 |
| **Postoperative SRT**  - No  - Yes | -  1.29 (0.56-2.97) | 0.54 | -  1.35 (0.57-3.18) | 0.49 | -  1.22 (0.51-2.94) | 0.64 | -  1.19 (0.50-2.81) | 0.68 |
| **Postoperative WBRT**  - No  - Yes | -  1.14 (0.46-2.79) | 0.77 | -  1.49 (0.60-3.64) | 0.38 | -  1.21 (0.51-2.88) | 0.66 | -  1.20 (0.50-2.86) | 0.67 |
| **Postoperative immunotherapy**  - No  - Yes | -  1.06 (0.44-2.56) | 0.88 | -  1.23 (0.48-3.14) | 0.66 | -  0.50 (0.21-1.16) | 0.10 | -  0.47 (0.20-1.08) | 0.075 |
| **Postoperative TT**  - No  - Yes | -  0.41 (0.15-1.06) | 0.068 | -  0.68 (0.27-1.66) | 0.40 | -  0.77 (0.30-1.95) | 0.59 | -  0.68 (0.25-1.83) | 0.45 |

**Supplemental Table 12. Multivariable Cox regression analysis for iPFS stratified by the presence of extracranial metastases at timepoint of BM surgery.**

BM, brain metastasis; ECOG, Eastern Cooperative Oncology Group; GTR, gross-total resection; iPFS, intracranial progression-free survival; SRT, stereotactic radiotherapy; TT, targeted therapy; WBRT, whole-brain radiotherapy. #Postoperative residual tumor volume values were log-transformed before fitting Cox regression model. *p value < 0.05.

|  | **Univariable Cox regression analysis (iPFS)** | | | |
| --- | --- | --- | --- | --- |
|  | **Postoperative immunotherapy** | | **No postoperative immunotherapy** | |
|  | HR (95%CI) | *P* value | HR (95%CI) | *P* value |
| **Gross-total resection (GTR)**  - No  - Yes | -  0.46 (0.26-0.83) | **0.010*** | -  0.42 (0.26-0.70) | **<0.001*** |
| **Postoperative residual tumor volume [cm^3^]#** | 1.11 (0.88-1.40) | 0.40 | 1.38 (1.12-1.69) | **0.002*** |
| **Age at BM surgery** | 1.00 (0.97-1.03) | >0.90 | 1.00 (0.98-1.03) | 0.70 |
| **Preoperative ECOG status**  - ECOG ≤1  - ECOG >1 | -  0.77 (0.24-2.48) | 0.70 | -  1.71 (0.89-3.26) | 0.11 |
| **Symptomatic BM**  - No  - Yes | -  1.86 (0.67-5.16) | 0.20 | -  1.19 (0.61-2.31) | 0.60 |
| **Number of BM**  - 1  - 2-5  - >5 | -  1.68 (0.94-2.99)  2.17 (0.98-4.78) | 0.077  0.056 | -  2.11 (1.34-3.33)  1.73 (0.81-3.27) | **0.001***  0.20 |
| **Extracranial metastases**  - No  - Yes | -  1.06 (0.62-1.82) | 0.80 | -  0.77 (0.50-1.20) | 0.20 |
| **Steroid intake at hospital discharge**  - No  - Yes | -  0.86 (0.43-1.71) | 0.70 | -  0.90 (0.57-1.41) | 0.60 |
| **Postoperative radiotherapy**  - No  - Yes | -  1.18 (0.29-4.88) | 0.80 | -  1.38 (0.75-2.54) | 0.30 |
| **Postoperative SRT**  - No  - Yes | -  0.84 (0.44-1.64) | 0.60 | -  1.20 (0.77-1.88) | 0.40 |
| **Postoperative WBRT**  - No  - Yes | -  1.71 (0.99-2.96) | 0.053 | -  1.05 (0.66-1.68) | 0.80 |
| **Postoperative TT**  - No  - Yes | -  0.78 (0.37-1.66) | 0.50 | -  0.89 (0.56-1.41) | 0.60 |

**Supplemental Table 13. Univariable Cox regression analysis for iPFS stratified by the administration of postoperative immunotherapy.**

BM, brain metastasis; ECOG, Eastern Cooperative Oncology Group; GTR, gross-total resection; iPFS, intracranial progression-free survival; SRT, stereotactic radiotherapy; TT, targeted therapy; WBRT, whole-brain radiotherapy. #Postoperative residual tumor volume values were log-transformed before fitting Cox regression model. *p value < 0.05.

|  | **Multivariable Cox regression analysis (iPFS)** | | | | | | | |
| --- | --- | --- | --- | --- | --- | --- | --- | --- |
|  | **Postoperative immunotherapy** | | | | **No postoperative immunotherapy** | | | |
|  | HR (95%CI) | *P* value | HR (95%CI) | *P* value | HR (95%CI) | *P* value | HR (95%CI) | *P* value |
| **Gross-total resection (GTR)**  - No  - Yes | -  0.53 (0.21-1.34) | 0.18 |  |  | -  0.45 (0.20-1.03) | 0.060 |  |  |
| **Postoperative residual tumor volume [cm^3^]#** |  |  | 0.88 (0.56-1.40) | 0.61 |  |  | 1.42 (0.99-2.04) | 0.054 |
| **Age at BM surgery** | 1.04 (1.00-1.09) | **0.040*** | 1.03 (0.99-1.08) | 0.076 | 1.01 (0.97-1.05) | 0.44 | 1.02 (0.98-1.06) | 0.17 |
| **Preoperative ECOG status**  - ECOG ≤1  - ECOG >1 | -  0.42 (0.10-1.66) | 0.21 | -  0.66 (0.15-2.76) | 0.57 | -  0.71 (0.31-1.63) | 0.43 | -  0.53 (0.21-1.30) | 0.16 |
| **Symptomatic BM**  - No  - Yes | -  4.19 (0.98-17.83) | 0.052 | -  3.65 (0.87-15.32) | 0.076 | -  1.35 (0.51-3.54) | 0.53 | -  1.48 (0.53-4.15) | 0.44 |
| **Number of BM**  - 1  - 2-5  - >5 | -  1.62 (0.69-3.80)  2.81 (0.82-9.58) | 0.25  0.098 | -  2.07 (0.97-4.42)  4.91 (1.46-16.50) | 0.058  **0.010*** | -  1.48 (0.68-3.23)  2.80 (0.66-11.77) | 0.31  0.15 | -  1.88 (0.92-3.85)  2.65 (0.57-12.29) | 0.081  0.21 |
| **Extracranial metastases**  - No  - Yes | -  1.06 (0.44-2.52) | 0.88 | -  1.29 (0.54-3.05) | 0.55 | -  0.52 (0.23-1.17) | 0.11 | -  0.58 (0.26-1.32) | 0.19 |
| **Steroid intake at hospital discharge**  - No  - Yes | -  0.58 (0.22-1.56) | 0.28 | -  0.54 (0.20-1.45) | 0.22 | -  0.50 (0.20-1.26) | 0.14 | -  0.62 (0.24-1.57) | 0.31 |
| **Postoperative SRT**  - No  - Yes | -  0.81 (0.32-2.02) | 0.65 | -  0.62 (0.23-1.66) | 0.34 | -  1.54 (0.68-3.44) | 0.29 | -  1.44 (0.65-3.19) | 0.35 |
| **Postoperative WBRT**  - No  - Yes | -  1.18 (0.53-2.66) | 0.67 | -  1.53 (0.72-3.28) | 0.26 | -  1.52 (0.65-3.56) | 0.32 | -  1.35 (0.55-3.30) | 0.50 |
| **Postoperative TT**  - No  - Yes | -  1.36 (0.35-5.19) | 0.64 | -  1.75 (0.48-6.33) | 0.38 | -  0.65 (0.30-1.42) | 0.28 | -  0.56 (0.24-1.31) | 0.18 |

**Supplemental Table 14. Multivariable Cox regression analysis for iPFS stratified by the administration of postoperative immunotherapy.**

BM, brain metastasis; ECOG, Eastern Cooperative Oncology Group; GTR, gross-total resection; iPFS, intracranial progression-free survival; SRT, stereotactic radiotherapy; TT, targeted therapy; WBRT, whole-brain radiotherapy. #Postoperative residual tumor volume values were log-transformed before fitting Cox regression model. *p value < 0.05.

|  | **Univariable Cox regression analysis (iPFS)** | | | |
| --- | --- | --- | --- | --- |
|  | **Postoperative TT** | | **No postoperative TT** | |
|  | HR (95%CI) | *P* value | HR (95%CI) | *P* value |
| **Gross-total resection (GTR)**  - No  - Yes | -  0.83 (0.36-1.92) | 0.70 | -  0.37 (0.24-0.57) | **<0.001*** |
| **Postoperative residual tumor volume [cm^3^]#** | 1.26 (0.84-1.87) | 0.30 | 1.20 (1.02-1.40) | **0.025*** |
| **Age at BM surgery** | 1.01 (0.98-1.04) | 0.60 | 1.00 (0.98-1.02) | >0.90 |
| **Preoperative ECOG status**  - ECOG ≤1  - ECOG >1 | -  5.34 (1.18-24.2) | **0.030*** | -  1.11 (0.61-2.02) | 0.70 |
| **Symptomatic BM**  - No  - Yes | -  0.75 (0.28-1.96) | 0.60 | -  1.71 (0.86-3.44) | 0.13 |
| **Number of BM**  - 1  - 2-5  - >5 | -  1.89 (0.87-4.09)  2.18 (0.79-5.99) | 0.11  0.13 | -  2.05 (1.37-3.07)  1.70 (0.88-3.26) | **<0.001***  0.11 |
| **Extracranial metastases**  - No  - Yes | -  0.60 (0.29-1.21) | 0.20 | -  0.93 (0.64-1.36) | 0.70 |
| **Steroid intake at hospital discharge**  - No  - Yes | -  0.55 (0.24-1.25) | 0.20 | -  0.97 (0.64-1.48) | 0.90 |
| **Postoperative radiotherapy**  - No  - Yes | -  1.92 (0.67-5.46) | 0.20 | -  1.04 (0.54-1.99) | >0.90 |
| **Postoperative SRT**  - No  - Yes | -  1.17 (0.56-2.42) | 0.70 | -  0.91 (0.59-1.41) | 0.70 |
| **Postoperative WBRT**  - No  - Yes | -  2.02 (0.92-4.42) | 0.078 | -  1.22 (0.82-1.82) | 0.30 |
| **Postoperative immunotherapy**  - No  - Yes | -  0.82 (0.37-1.83) | 0.60 | -  0.83 (0.57-1.21) | 0.30 |

**Supplemental Table 15. Univariable Cox regression analysis for iPFS stratified by the administration of postoperative TT.**

BM, brain metastasis; ECOG, Eastern Cooperative Oncology Group; GTR, gross-total resection; iPFS, intracranial progression-free survival; SRT, stereotactic radiotherapy; TT, targeted therapy; WBRT, whole-brain radiotherapy. #Postoperative residual tumor volume values were log-transformed before fitting Cox regression model. *p value < 0.05.

|  | **Multivariable Cox regression analysis (iPFS)** | | | | | | | |
| --- | --- | --- | --- | --- | --- | --- | --- | --- |
|  | **Postoperative TT** | | | | **No postoperative TT** | | | |
|  | HR (95%CI) | *P* value | HR (95%CI) | *P* value | HR (95%CI) | *P* value | HR (95%CI) | *P* value |
| **Gross-total resection (GTR)**  - No  - Yes | -  2.72 (0.63-11.66) | 0.17 |  |  | -  0.39 (0.20-0.74) | **0.004*** |  |  |
| **Postoperative residual tumor volume [cm^3^]#** |  |  | 1.30 (0.65-2.57) | 0.45 |  |  | 1.16 (0.86-1.56) | 0.20 |
| **Age at BM surgery** | 1.06 (1.00-1.13) | **0.033*** | 1.09 (1.00-1.19) | **0.031*** | 1.01 (0.98-1.05) | 0.27 | 1.02 (0.99-1.05) | 0.13 |
| **Preoperative ECOG status**  - ECOG ≤1  - ECOG >1 | -  10.29 (1.24-85.16) | **0.030*** | -  4.88 (0.82-29.02) | 0.080 | -  0.48 (0.23-1.02) | 0.056 | -  0.45 (0.20-1.01) | 0.053 |
| **Symptomatic BM**  - No  - Yes | -  1.57 (0.30-8.19) | 0.59 | -  1.53 (0.25-9.14) | 0.63 | -  2.81 (1.04-7.59) | **0.041*** | -  2.22 (0.83-5.90) | 0.10 |
| **Number of BM**  - 1  - 2-5  - >5 | -  2.75 (0.61-12.23)  31.93 (3.49-291.64) | 0.18  **0.002*** | -  1.29 (0.32-5.11)  16.54 (2.18-125.47) | 0.71  **0.006*** | -  1.46 (0.77-2.77)  1.82 (0.64-5.20) | 0.24  0.25 | -  2.17 (1.22-3.87)  3.02 (1.06-8.57) | **0.008***  **0.037*** |
| **Extracranial metastases**  - No  - Yes | -  0.14 (0.035-0.55) | **0.005*** | -  0.16 (0.03-0.69) | **0.014*** | -  0.92 (0.51-1.68) | 0.80 | -  0.91 (0.50-1.64) | 0.76 |
| **Steroid intake at hospital discharge**  - No  - Yes | -  0.073 (0.011-0.48) | **0.006*** | -  0.10 (0.01-0.69) | **0.018*** | -  0.71 (0.34-1.47) | 0.36 | -  0.79 (0.38-1.61) | 0.52 |
| **Postoperative SRT**  - No  - Yes | -  1.26 (0.38-4.08) | 0.69 | -  1.27 (0.38-4.26) | 0.69 | -  0.91 (0.47-1.76) | 0.78 | -  0.92 (0.46-1.85) | 0.82 |
| **Postoperative WBRT**  - No  - Yes | -  1.11 (0.19-6.18) | 0.90 | -  1.08 (0.19-6.06) | 0.92 | -  1.21 (0.64-2.27) | 0.54 | -  1.44 (0.76-2.72) | 0.26 |
| **Postoperative immunotherapy**  - No  - Yes | -  5.23 (0.81-33.72) | 0.081 | -  4.10 (0.66-25.18) | 0.12 | -  0.47 (0.25-0.87) | **0.017*** | -  0.47 (0.25-0.85) | **0.013*** |

**Supplemental Table 16. Multivariable Cox regression analysis for iPFS stratified by the administration of postoperative TT.**

BM, brain metastasis; ECOG, Eastern Cooperative Oncology Group; GTR, gross-total resection; iPFS, intracranial progression-free survival; SRT, stereotactic radiotherapy; TT, targeted therapy; WBRT, whole-brain radiotherapy. #Postoperative residual tumor volume values were log-transformed before fitting Cox regression model. *p value < 0.05.

|  | **Multivariable Cox regression analysis in non-GTR subgroup** | | | |
| --- | --- | --- | --- | --- |
|  | **OS** | | **iPFS** | |
|  | HR (95%CI) | *P* value | HR (95%CI) | *P* value |
| **Postoperative residual tumor volume [cm^3^]#** | 0.87 (0.56-1.33) | 0.53 | 0.87 (0.56-1.33) | 0.75 |
| **Age at BM surgery** | 1.00 (0.97-1.03) | 0.71 | 1.00 (0.97-1.03) | 0.072 |
| **Preoperative ECOG status**  - ECOG ≤1  - ECOG >1 | -  2.96 (1.38-6.34) | **0.005*** | -  2.96 (1.38-6.34) | 0.40 |
| **Symptomatic BM**  - No  - Yes | -  0.55 (0.24-1.25) | 0.15 | -  0.55 (0.24-1.25) | 0.28 |
| **Number of BM**  - 1  - 2-5  - >5 | -  0.99 (0.51-1.89)  0.86 (0.36-2.03) | 0.98  0.73 | -  0.99 (0.51-1.89)  0.86 (0.36-2.03) | 0.53  0.10 |
| **Extracranial metastases**  - No  - Yes | -  2.33 (1.20-4.52) | **0.012*** | -  2.33 (1.20-4.52) | 0.76 |
| **Steroid intake at hospital discharge**  - No  - Yes | -  0.71 (0.31-1.64) | 0.43 | -  0.71 (0.31-1.64) | 0.72 |
| **Postoperative SRT**  - No  - Yes | -  0.99 (0.47-2.07) | 0.99 | -  0.99 (0.47-2.07) | 0.82 |
| **Postoperative WBRT**  - No  - Yes | -  1.68 (0.84-3.37) | 0.80 | -  1.68 (0.84-3.37) | 0.62 |
| **Postoperative immunotherapy**  - No  - Yes | -  0.44 (0.23-0.85) | **0.014*** | -  0.44 (0.23-0.85) | 0.10 |
| **Postoperative TT**  - No  - Yes | -  0.35 (0.17-0.72) | **0.004*** | -  0.35 (0.17-0.72) | **0.045*** |

**Supplemental Table 17. Multivariable Cox regression analysis for OS restricted to the non-GTR subgroup.**

BM, brain metastasis; ECOG, Eastern Cooperative Oncology Group; GTR, gross-total resection; iPFS, intracranial progression-free survival; OS, intracranial progression-free survival; SRT, stereotactic radiotherapy; TT, targeted therapy; WBRT, whole-brain radiotherapy. #Postoperative residual tumor volume values were log-transformed before fitting Cox regression model. *p value < 0.05.


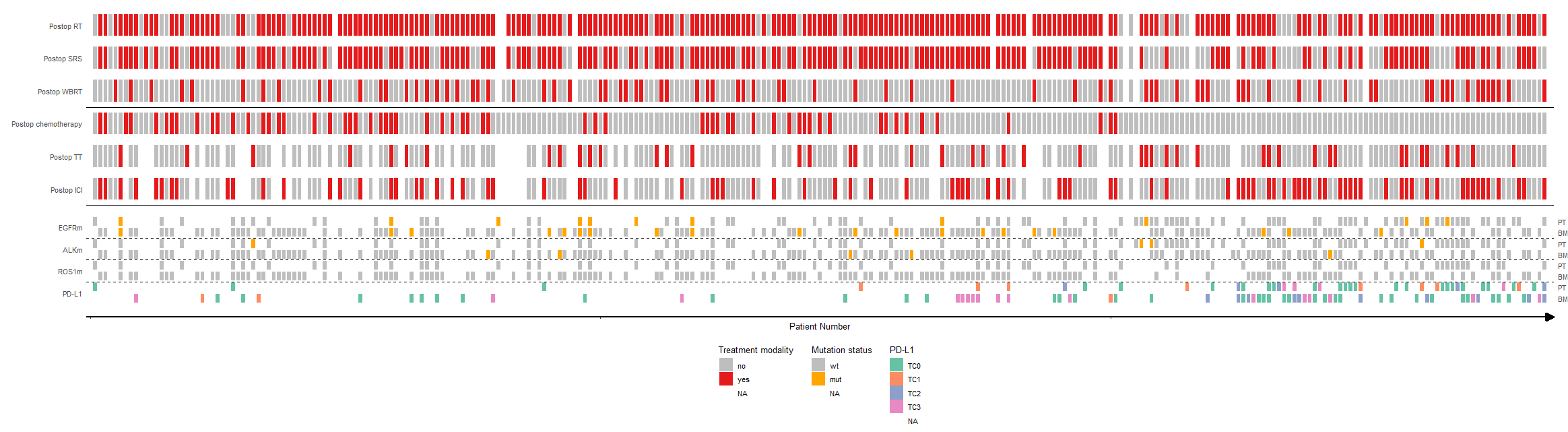


**Supplemental Figure 1. Heatmap illustrating distinct patients with their postoperative treatment modalities and molecular tumor status.**

ALK, anaplastic lymphoma kinase; BM, brain metastasis; EGFR, epidermal growth factor receptor; ICI, immune checkpoint inhibitor; NA, not applicable; PT, primary tumor; RT, radiotherapy; SRS, stereotactic radiosurgery; TC, tumor cell score (TC0: <1%; TC1: 1-4%; TC2: 5-49%; TC3: ≥50%); TT, targeted therapy; WBRT, whole-brain radiotherapy; WT, wild type.


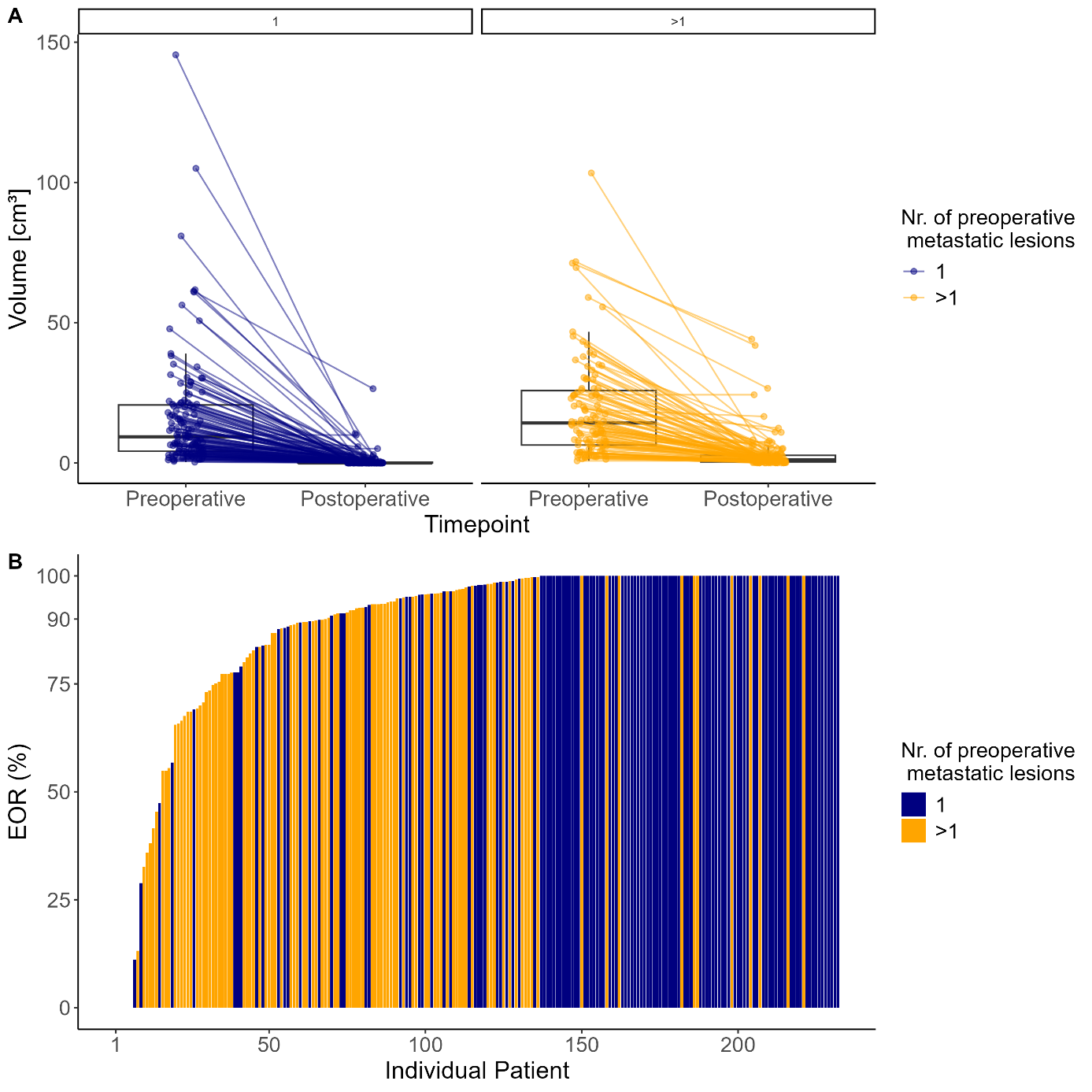


**Supplemental Figure 2. Pre- and postoperative intracranial tumor volumes and extent of resection (EOR) stratified by number of brain metastases.**

(A) Pre- and postoperative tumor volumes for each patient, stratified by number of preoperative intracranial lesions (1 vs. >1). Lines connect paired volumes, and boxplots indicate group-level distributions.

(B) Extent of resection (EOR, %) across individual patients, ordered by EOR magnitude and stratified by number of preoperative lesions (1 vs. >1). The plot illustrates a high frequency of gross-total resections (GTR, i.e. EOR = 100%) in patients with solitary brain metastases.


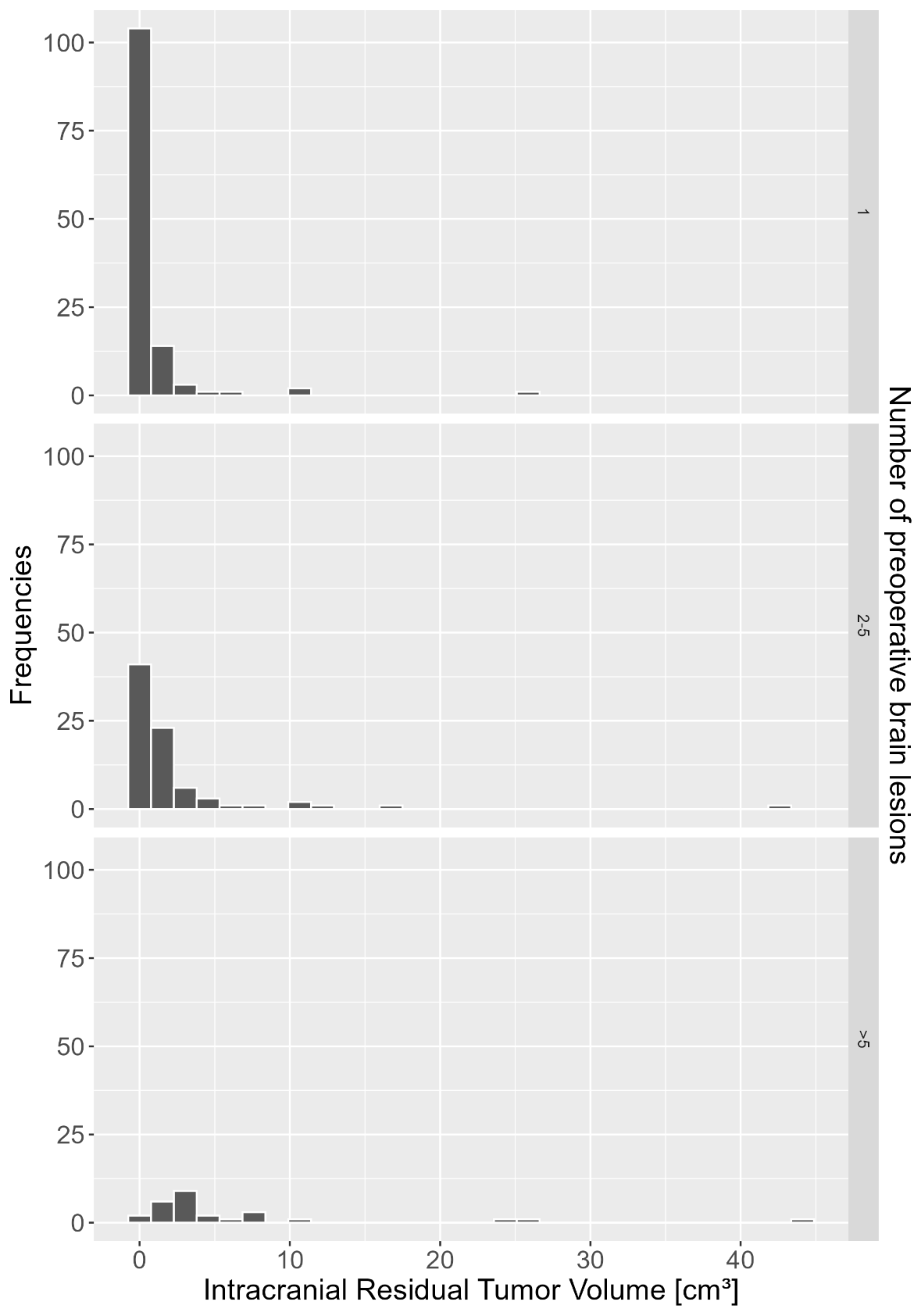


**Supplemental Figure 3. Distribution of postoperative intracranial residual tumor volume stratified by number of brain metastases.**

Histogram of postoperative intracranial residual tumor volume (cm^3^) stratified by the number of preoperative brain metastases (single, 2–5, and >5 lesions). Across all strata, the distribution is markedly right-skewed, with most patients exhibiting minimal to no measurable residual disease and a small subset displaying substantially higher residual volumes. The pattern is consistent across groups, although patients with >5 lesions show slightly higher residual volumes overall.


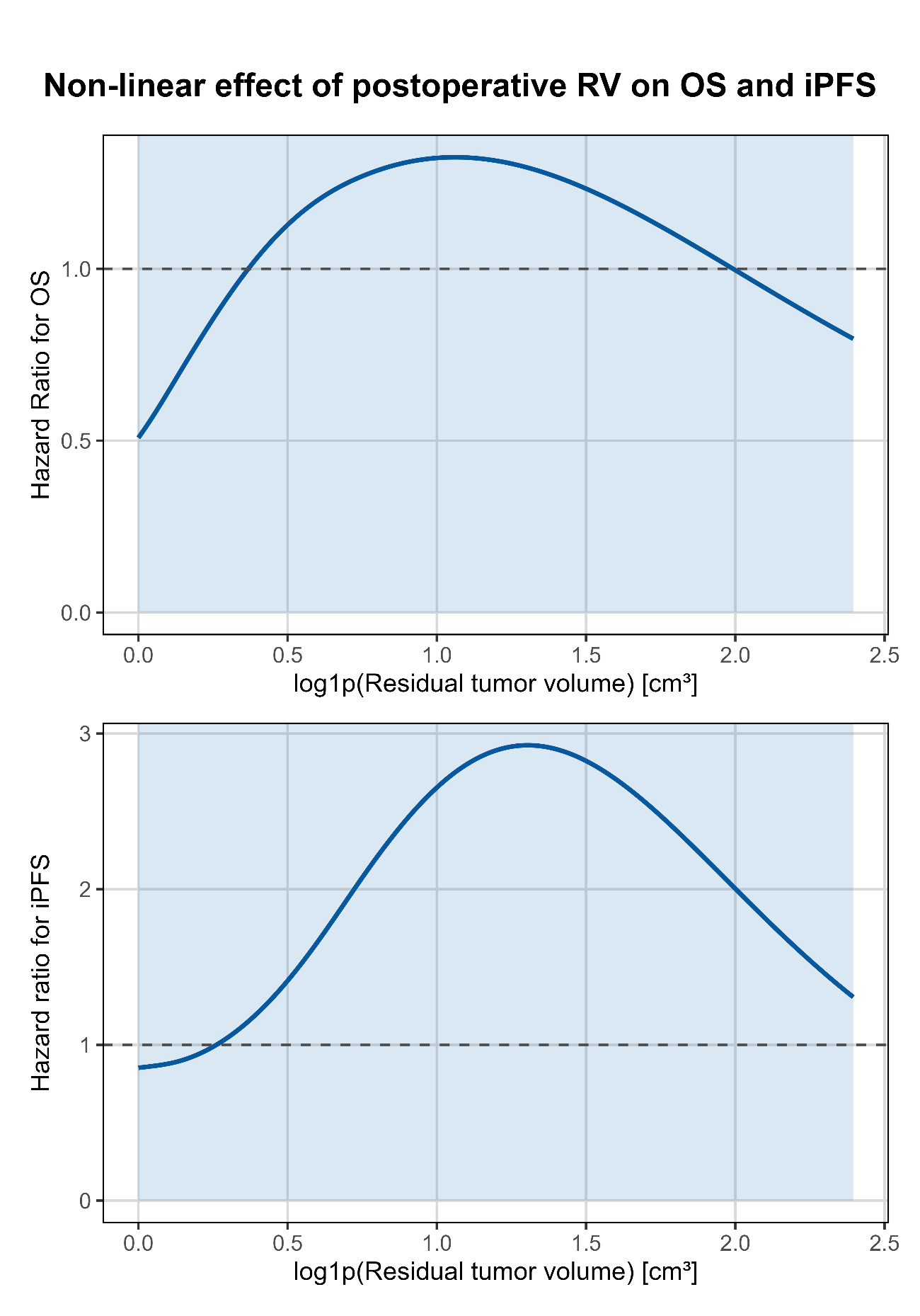


**Supplemental Figure 4. Non-linear effect of postoperative residual tumor volume on overall survival and intracranial progression-free survival.**

This figure illustrates the estimated non-linear association between postoperative residual tumor volume (RV) and survival outcomes using restricted cubic spline Cox regression models. The upper panel shows the hazard ratio (HR) for overall survival (OS), and the lower panel shows the HR for intracranial progression-free survival (iPFS). Solid blue lines represent the spline-predicted HR across the range of observed residual volumes; shaded ribbons indicate the 95% confidence intervals. The dashed horizontal line denotes HR = 1 (no effect).
